# PEGylation of indium phosphide quantum dots prevents quantum dot mediated platelet activation†

**DOI:** 10.1039/d4tb01334d

**Published:** 2024-12-05

**Authors:** Leigh Naylor-Adamson, Thomas W. Price, Zoe Booth, Sophie V. L. Leonard, Juan Gallo, Le Duc Tung, Stanley Harvell-Smith, Nguyen Thi Kim Thanh, Zabeada Aslam, David Allsup, Nicole Hondow, Thomas Chamberlain, Jürgen E. Schneider, Khalid Naseem, Jean-Sebastien G. Bouillard, Graeme J. Stasiuk, Simon D. J. Calaminus

**Affiliations:** a Biomedical Institute for Multimorbidity, Hull York Medical School, University of Hull Hull HU6 7RX UK simon.calaminus@hyms.ac.uk; b Department of Imaging Chemistry and Biology, School of Biomedical Engineering and Imaging Sciences, King's College London London SE1 7EH UK graeme.stasiuk@kcl.ac.uk; c Advanced Magnetic Theranostic Nanostructures Lab, International Iberian Nanotechnology Laboratory, Av. Mestre José Veiga Braga 4715-330 Portugal; d UCL Healthcare Biomagnetics and Nanomaterials Laboratories 21 Albemarle Street London W1S 4BS UK; e Biophysics Group, Department of Physics & Astronomy University College London, Gower Street London WC1E 6BT UK; f Leeds Electron Microscopy and Spectroscopy Centre, LEMAS, The Bragg Centre for Materials Research, Faculty of Engineering and Physical Sciences, University of Leeds LS2 9JT UK; g Institute of Process Research and Development, School of Chemistry, University of Leeds Leeds LS2 9JT UK; h School of Medicine, University of Leeds Leeds LS2 9JT UK; i Leeds Institute of Cardiovascular and Metabolic Medicine, University of Leeds Leeds LS2 9JT UK; j Department of Physics and Mathematics, Nano^3^ Research grouping - Nanophotonics group, G. W. Gray Centre for Advanced Materials, University of Hull Cottingham Road Hull HU6 7RX UK

## Abstract

Quantum dots (QDs) are semiconducting inorganic nanocrystals, that have garnered interest in biological and medical spheres due, to their potential benefits in biomedical imaging and drug-delivery systems. Indium phosphide QDs shelled with zinc sulphide (InP/ZnS) are viewed as more biocompatible than previous heavy metal based QDs. However, little is known about how InP/ZnS QDs affect a key blood cell, the platelet. Understanding how platelets interact with QDs is critical as unwanted activation can lead to pathological thrombus formation. Herein, we demonstrate PEGylation of InP/ZnS QDs coated with lipoic acid (QD-LA) or coated with penicillamine (QD-Pen) surface ligands induced markedly less platelet aggregation, platelet–QD interactions, integrin activation, alpha granule secretion and restored platelet spreading in washed platelets in comparison to their non-PEGylated counterparts. Furthermore, in whole blood, PEGylation of QDs reduced the number of QDs in the thrombus, thereby helping to minimise the chance of dysfunctional thrombus formation. Overall, we show that QD PEGylation is important to help prevent QD mediated platelet activation. In combination with the most biocompatible coating, PEGylation markedly reduced platelet activation, widening the concentrations at which QDs were viable for development as potential drug delivery or imaging agents.

## Abstract 2

Quantum dots (QDs) are semiconducting inorganic nanocrystals, that have garnered interest in biological and medical spheres, due to their potential benefits in biomedical imaging and drug-delivery systems.

## Graphical abstract

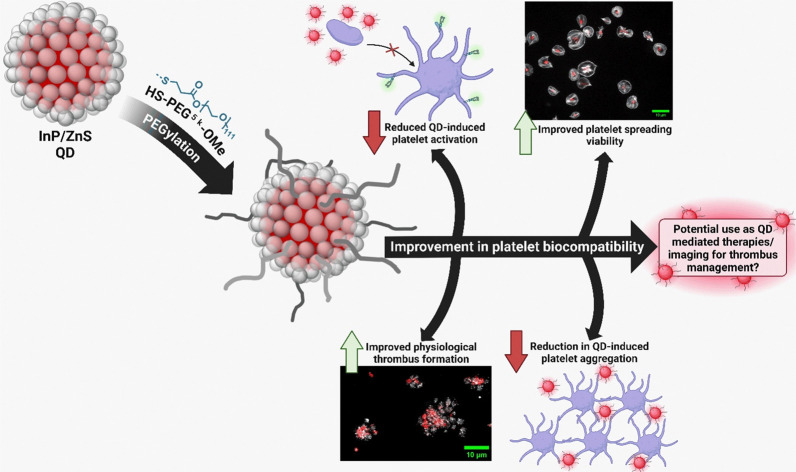

## Introduction

Platelets play a critical role in the pathogenesis of multiple diseases, including cancer, diabetes, and cardiovascular disease (CVD). Inappropriate platelet activation can cause the formation of pathological thrombi, which can lead to heart attacks or strokes.^1^ Effective identification of thrombi *in vivo* is potentially of clinical benefit as such visualisation could aid in the diagnosis of disease states.

At present, there are a range of clinically available imaging modalities to identify thrombi such as computed tomography (CT), magnetic resonance imaging (MRI) and ultrasound.^2^ However, such modalities are designed to identify hypoperfusion of affected tissues rather than the thrombus. Nanotechnological devices, such as quantum dots (QDs), are the subject of investigation as aides to clinical imaging techniques. QDs are semiconducting nanocrystals with a size-dependent tuneable emission. The core/shell structured QDs historically contain heavy metals in their cores.^3,4^ These cores can be made up of elements from groups II–VI, group IV, and groups III–V. Importantly, to improve biocompatibility and fluorescent properties, QDs can be shelled with elements from groups II–VI. QDs represent favourable fluorescent probes for biomedical imaging due to their photostability, large Stokes shift, high brightness, and extended excited state lifetimes.^4^ Indeed, QDs have been previously used to image atherosclerosis *in vivo*.^5–7^ There is also the possibility of multimodal imaging capacity due to the potential combination of QDs with radioisotopes for positron emission tomography (PET) or single photon emission computed tomography (SPECT), or contrast agents for MRI.^8–10^ In addition, QDs have been used as drug delivery systems.^11,12^

In the context of platelets and coagulation, cadmium (Cd) based QDs have been the most intensively investigated. CdTe/ZnS QDs have been found to interact with, and cause conformation changes in, the clotting proteins fibrinogen, plasminogen, and prothrombin.^13^ In addition, the size and surface chemistry of the QDs is also relevant with smaller QDs associated with activation of the coagulation cascade.^14^

It has been demonstrated that Cd-based QDs can activate platelets with resultant aggregation. Smaller CdTe QDs cause activation at lower concentrations than larger QDs.^15^ CdSe/ZnS QDs functionalised with carboxyl groups cause thrombosis in a dose-dependent manner, which was not observed with amine-functionalised QDs, potentially due to differing zeta potentials. These QDs did not result in platelet aggregation, with observed platelet activation being secondary to activation of the coagulation cascade.^16^

Previously, we have investigated InP/ZnS QDs as a biocompatible alternative to toxic Cd based QDs. The InP/ZnS QDs were coated with either thioglycolic acid (TGA), lipoic acid (LA), penicillamine (Pen), or glutathione (GSH) before being phase transferred to biocompatible aqueous media. We found that platelet–QD activation of platelets was coating-dependent, with QDs coated with TGA and Pen surface coatings having greater platelet biocompatibility.^17^

However, although more biocompatible than Cd-based QDs, significant further work was needed to further improve the biocompatibility of InP/ZnS QDs. Such an improvement in biocompatibility would reduce dysregulation of platelet activation and therefore prevent unwanted thrombus formation. To reduce InP/ZnS QD mediated platelet activation, QDs were coated with polyethylene glycol (PEG). Alongside improved biocompatibility, PEG has been shown to improve pharmacokinetic behaviour of proteins and small molecule drugs.^18^ PEGylation of QDs decreased the activation of platelets when compared to non-PEGylated QDs, both in terms of platelet aggregation as well as in respect of platelet activation markers. In addition, PEGylation of QDs re-established the ability of platelets to spread on fibrinogen and collagen (another marker of platelet activation), and reduced interaction of QDs with thrombi in whole blood.

Overall, PEGylation reduces QD mediated platelet activation, further demonstrating the potential of InP/ZnS QDs as potential drug delivery or imaging agents for use *in vivo*.

## Experimental methods

QD samples were prepared and analysed as previously described.^17,19^ Additional experimental information is present in the ESI.†

### Ethics and donor recruitment

Written informed consent was obtained from drug-free, healthy volunteers. Ethical approvals were obtained from the Hull York Medical School Ethics Committee, University of Hull, UK and the National Health Service Health Research Authority, study number 21/SC/0215.

### Preparation of blood

For washed platelet preparation, whole blood was collected from healthy donors into acid citrate dextrose (ACD) vacutainers (BD) and prepared as previously described.^17^

### Light transmission aggregometry (LTA)

Washed platelets (2.5 × 10^8^ mL^−1^) were monitored at 37 °C in 1200 rpm stirring conditions using a CHRONOLOG Model 490 4 + 4 aggregometer with AGGRO/LINK Opti8 software (Chronolog, USA). Platelets were incubated with 10, 30, or 100 nM QDs or with a vehicle control (Tyrode's buffer) for 10 minutes before stimulation with 0.1 U mL^−1^ thrombin or 3 μg mL^−1^ collagen and monitored for a further 10 minutes.

### Platelet spreading

Coverslips were coated with either 100 μg mL^−1^ fibrinogen or 100 μg mL^−1^ collagen overnight at 4 °C. Washed platelets (2 × 10^7^ mL^−1^) were spread on these coverslips with either 10, 30, or 100 nM QDs for 25 minutes at 37 °C. Coverslips then fixed, mounted and imaged as previously described.^17^

### Thrombus formation under flow

Microfluidic biochips (Vena8 Endothelial+ Microfluid Biochips, Cellix: Dublin, Ireland) were pre-coated with 100 μg mL^−1^ collagen overnight before blocking with 5 mg mL^−1^ BSA for 15 minutes. Whole blood was collected in 40 μM PPACK and stained with 10 μM DiOC_6_. 100 nM of QDs was added to whole blood for 20 minutes before being flowed over the chip at 1000 s^−1^ arterial shear for 4 minutes. The chip was then fixed and imaged as previously described.^20^

### Flow cytometry

Washed platelets (1 × 10^7^ mL^−1^) were incubated for 5 minutes with relevant antibodies and relative isotype controls as previously described.^17^ An unstained sample was also included. Relevant QDs at either 30 or 100 nM were incubated with platelets for 20 minutes before stimulation with 0.1 U mL^−1^ thrombin for 10 minutes. Platelets were fixed and analysed as previously published.^17^ The gating strategy is depicted in Fig. S1 (ESI†) to identify % platelets positive for each stain or QD. Mean fluorescence intensity (MFI) was identified from the CD42b gate for each stain or QD.

### Statistical analysis

All statistical tests were performed using GraphPad Prism 8.0.1 software (GraphPad, San Diego, USA). Data is presented as mean ± standard deviation (SD). Analysis of normality was assessed *via* Shapiro–Wilk and Kolmogorov–Smirnov tests. For parametric data, groups were analysed by one-way analysis of variance (ANOVA), followed by a *post hoc* test of Tukey's multiple comparison test. For comparison between PEGylated and non-PEGylated QDs, data was analysed by two-way ANOVA, followed by a *post hoc* test of Sidak's multiple comparison test. *P* < 0.05 was considered significant.

## Results

### Characteristics of QDs after PEGylation

InP/ZnS QDs were prepared in line with published procedures.^17,19^ The InP cores had a size of 2.86 ± 0.5 nm (Fig. S2, ESI†), with a cubic InP structure (Fig. S3, ESI†). Energy dispersive X-ray spectroscopy (EDX) gives an In : P ratio of 49 : 51 for the core nanoparticles (Table S1, ESI†). Following shelling with ZnS, the QD size increased to 3.8 ± 0.6 nm (Fig. S4, ESI†) and cubic ZnS can be observed in the PXRD pattern obtained (Fig. S3, ESI†).

**Fig. 1:**
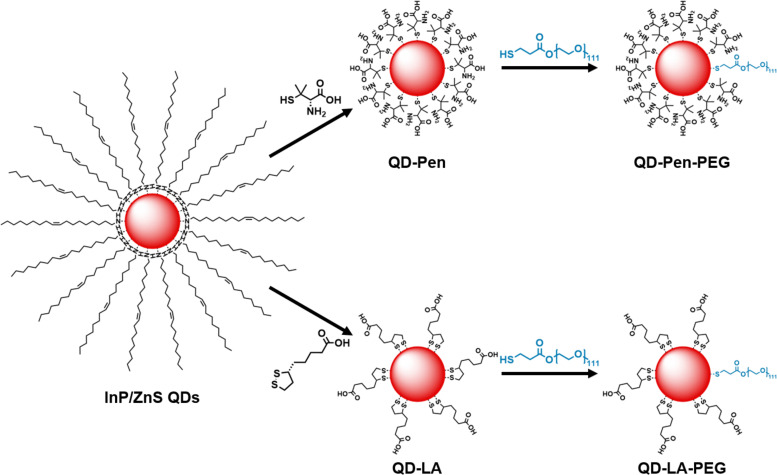
Preparation of QD formulations. Schematic of the synthetic procedures for preparation of QD-Pen, QD-LA, QD-Pen-PEG, and QD-LA-PEG from InP/ZnS QDs. Please note, ligand numbers and ratios are not representative.

The hydrophobic ligands on the surface of the InP/ZnS QDs were exchanged for hydrophilic ligands to promote dispersibility in water (Fig. 1).^21^ This was undertaken with two different thiol species; Pen, or LA, in a biphasic phase transfer reaction.

A slight decrease in the nanoparticle diameter is observed following phase transfer *D*_TEM_(QD-Pen) = 2.63 ± 0.5 nm and *D*_TEM_(QD-LA) = 3.29 ± 0.6 nm (Fig. S5 and S6, ESI†), and both the cubic InP and ZnS phases are retained (Fig. S3, ESI†). Thermogravimetric analysis of QD-Pen (Fig. S7A, ESI†) and QD-LA (Fig. S7B, ESI†) gives inorganic contents of 62.5% and 80.6% respectively. The presence of each ligand can also be confirmed by FT-IR spectroscopy, with a key stretch at 1600 cm^−1^ being identifiable in the QD-Pen sample (Fig. S8, ESI†), and at 2865, 2920 1570, and 1400 cm^−1^ being observed for QD-LA (Fig. S9, ESI†).

QD-LA has a more compact hydrodynamic diameter (*D*_H_(QD-LA) = 7.8 ± 0.5 nm), in comparison to QD-Pen (*D*_H_(QD-Pen) = 29.6 ± 0.9 nm, Fig. S10A and B, ESI†); this is also accompanied by a more negative zeta potential (*ζ*(QD-LA) = −27.3 ± 1.39 mV (Fig. S11A, ESI†), *ζ*(QD-Pen) = −14.7 ± 0.31 (Fig. S11B, ESI†)).

Slight differences were observed in the optical properties of QD-Pen and QD-LA; a minor red shift in both 1st excitonic peak and emission maxima were observed for QD-LA (Table S2 and Fig. S12, S13, ESI†). Furthermore, the photoluminescent quantum yield (*φ*) for QD-LA was greater (*φ*(QD-LA) = 23.6%, *φ*(QD-PEN) = 7.4%) and the emission lifetime was longer (〈*τ*〉_amp_(QD-LA) = 76.8 ± 5.9 ns, 〈*τ*〉_amp_(QD-Pen) = 58.0 ± 3.4 ns, Table S2 and Fig. S14, S15, ESI†).

O-[2-(3-mercaptopropionylamino)ethyl]-O′-methylpolyethylene glycol (HS-PEG^5k^-OMe) ligands were added to the QDs through a ligand exchange reaction yielding QD-Pen-PEG and QD-LA-PEG (Fig. 1). QD-Pen-PEG were determined to have a similar size to QD-Pen (*D*_TEM_QD-Pen-PEG = 2.82 ± 0.7 nm, Fig. S16, ESI†). In contrast, QD-LA-PEG were substantially smaller than QD-LA (*D*_TEM_(QD-LA-PEG) = 1.99 ± 0.4 nm, Fig. S17, ESI†). The cubic InP and ZnS structures are retained upon ligand exchange in both cases (Fig. S3, ESI†). Evidence for addition of the HS-PEG^5k^-OMe ligands can be seen through FT-IR spectroscopy; the appearance of stretches at 2865 cm^−1^ and 1100 cm^−1^ correspond to the presence of HS-PEG^5k^-OMe. However, stretches corresponding to Pen at 1600 cm^−1^ and LA at 2920, 2865, 1570, and 1400 cm^−1^ are still observed (Fig. S8 and S9, ESI†). These samples showed a reduced inorganic mass content; 59.4% of QD-Pen-PEG, and 59.3% of QD-LA-PEG (Fig. S7C and D, ESI†).

QD-Pen-PEG have a smaller hydrodynamic diameter than QD-Pen (*D*_H_(QD-Pen-PEG) = 24.63 ± 0.45 nm, Table S2 and Fig. S10C, ESI†); in contrast QD-LA-PEG display an increased hydrodynamic diameter than QD-LA (*D*_H_(QD-LA-PEG) = 12.56 ± 0.72 nm, Table S2 and Fig. S10D, ESI†). However, in both cases, zeta potential was reduced following PEGylation (Table S2 and Fig. S11C, D, ESI†).

The optical properties were broadly retained upon PEGylation (Table S3, ESI†); QD-Pen-PEG displays a 12 nm red shift in emission maximum (Fig. S18 and S19, ESI†), and in both cases *φ* increases following PEGylation to 15.7% for QD-Pen-PEG and 26.7% for QD-LA-PEG. QD-Pen-PEG has an extended lifetime (〈*τ*〉_amp_QD-Pen-PEG = 62.9 ± 3.5 ns, Table S3, ESI†), QD-LA-PEG has a reduced lifetime in comparison to QD-LA (〈*τ*〉_amp_QD-LA-PEG = 72.1 ± 3.1 ns, Table S3, ESI†).

It is clear that some changes to the physical properties of these nanomaterials are observed following the addition of HS-PEG^5k^-OMe ligands. To investigate the impact the addition of HS-PEG^5k^-OMe ligands to the surface of the QDs had on their interactions with platelets, QD-Pen, QD-LA, QD-Pen-PEG, and QD-LA-PEG were all tested using a panel of assays, allowing for comparisons between ligands, and following the addition of HS-PEG^5k^-OMe.

### PEGylation of QD-LA and QD-Pen markedly reduces QD-mediated platelet activation

Previously, we have determined that InP/ZnS QDs coated with Pen or LA caused platelet aggregation at 100 nM.^17^ A significant increase in platelet aggregation was still observed at 10 nM QD-LA, whilst this was not the case for QD-Pen. As such, we investigated the effects of PEGylation of QDs on platelet activation within this concentration range.

**Fig. 2:**
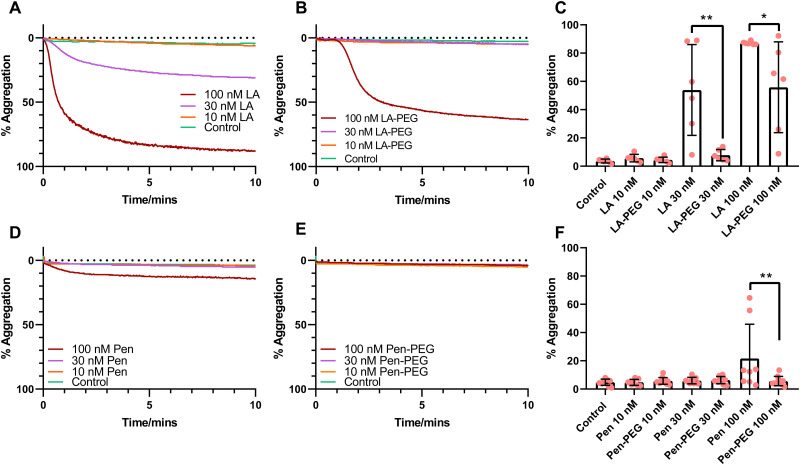
PEGylation of QDs markedly reduces QD-mediated platelet aggregation. Washed platelets at 2.5 × 10^8^ mL^−1^ were incubated with either (A) QD-LA (*n* = 6) and (B) QD-LA-PEG (*n* = 6), or (D) QD-Pen (*n* = 8) and (E) QD-Pen-PEG (*n* = 11) at either 10, 30 or 100 nM in stirring conditions at 37 °C for 10 min whilst aggregation was monitored, with the representative traces shown. Maximal aggregation response after 8 min is shown in (C) and (F). Data is shown as mean ± SD. Statistical significance was calculated using a two-way ANOVA followed by a Sidak's multiple comparison *post hoc* test. *p ≤ 0.05, **p ≤ 0.01, ***p ≤ 0.001.

Light transmission aggregometry (LTA) was performed to assess platelet aggregation in response to QDs. Washed platelets were incubated with QD-LA, QD-LA-PEG, QD-Pen, or QD-Pen-PEG (10–100 nM) for 10 minutes (Fig. 2). As previously observed, both 30 and 100 nM QD-LA caused platelet aggregation.^17^ Interestingly, PEGylation of QD-LA caused two effects: at 30 nM QD-LA-PEG caused no aggregation, whilst at 100 nM QD-LA-PEG caused a delayed induction of aggregation, as well as a reduced overall maximum aggregation compared to 100 nM QD-LA. This profile was mimicked with QD-Pen, with QD-Pen-PEG no longer inducing aggregation in comparison to QD-Pen. Additionally, PEGylation even reduced the response to 200 nM QD-Pen with a marked delay in aggregation induced by the QD-Pen-PEG in comparison to QD-Pen, even if both induced a similar level of aggregation after 10 minutes (Fig. S20, ESI†). Overall, this shows that PEGylation of QD is effective at reducing QD induced platelet aggregation.

To confirm the platelet aggregation results, flow cytometric analysis was used to investigate P-selectin (a marker of platelet secretion) and PAC1 (a marker of activated integrin α_IIb_β_3_), a critical component of platelet aggregation. Platelets were incubated with QD-LA, QD-LA-PEG, QD-Pen, or QD-Pen-PEG for 20 min and stained for P-selectin and PAC-1. Platelets were first gated by size and then for the platelet specific marker CD42. The gating strategy for each antibody and QD used can be found in Fig. S1 (ESI†).

**Fig. 3:**
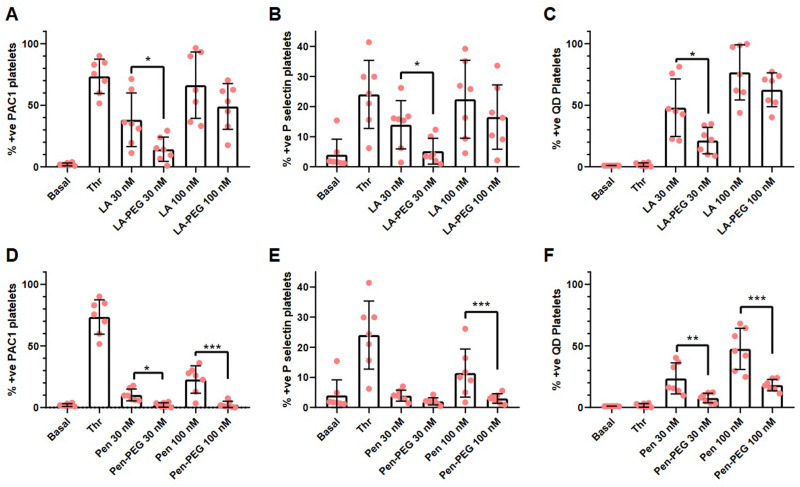
PEGylation of QDs markedly reduces QD-mediated increases in platelet activation. Washed platelets at 1 × 10^7^ mL^−1^ were incubated with monoclonal antibodies against P-selectin, CD42b, and PAC1 for 5 min before the addition of either QD-LA, QD-LA-PEG, QD-Pen, or QD-Pen-PEG at either 30 nM or 100 nM for 20 min before fixation with PFA and analysis by flow cytometry. Number of platelets positive for (A) and (D) PAC1, (B) and (E) P-selectin, and (C) and (F) QDs are shown (*n* = 7). Data is shown as mean ± SD. Statistical significance was calculated using a two-way ANOVA followed by a Sidak's multiple comparison *post hoc* test. *p ≤ 0.05, **p ≤ 0.01, ***p ≤ 0.001.

We observed that QD-LA caused a high level of platelet activation in a dose-dependent manner (Fig. 3). 100 nM QD-LA caused similar levels of platelet activation as thrombin stimulation in terms of P-selectin and PAC1 expression. PEGylation of QD-LA showed a decrease in platelet activation markers, with 30 nM QD-LA-PEG having similar levels of PAC1 and P-selectin expression as basal platelets and significantly lower than thrombin activated platelets. In agreement with the aggregation results, QD-Pen induced less platelet activation than QD-LA, with QD-Pen PAC1 and P-selectin expression being significantly lower in comparison to thrombin stimulation. Again, PEGylation of QD-Pen resulted in platelet expression levels of PAC1 and P-selectin being similar to basal platelets.

The self-fluorescence of the QDs also facilitated the analysis of these elements by flow cytometry. The number of platelets positive for QD-LA or QD-Pen increased in a dose-dependent manner. This interaction is reduced by PEGylation. The trends in platelet activation and QD binding is also reflected in the Mean Fluorescent Intensities (MFIs, Fig. S22, ESI†). Overall, this shows that PEGylation of QDs is effective in lowering the extent of QD-mediated platelet activation.

### Thrombin-mediated platelet activation is re-established by PEGylation of QDs

**Fig. 4:**
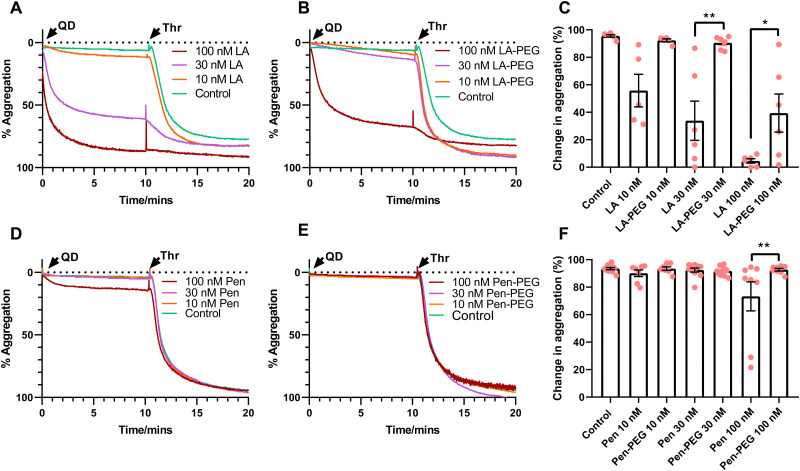
PEGylation of QDs preserve platelets’ ability to aggregate to thrombin. Washed platelets (2.5 × 10^8^ mL^−1^) were incubated at 37 °C in stirring conditions with either (A) QD-LA (*n* = 6) and (B) QD-LA-PEG (*n* = 6), or (D) QD-Pen (*n* = 8) and (E) QD-Pen-PEG (*n* = 11) at either 10, 30 or 100 nM for 10 min before being stimulated with 0.1 U mL^−1^ thrombin (Thr). Aggregation was monitored with representative traces shown. (C) and (F) show change in levels of aggregation following the addition of thrombin. Data is shown as mean ± SD. Statistical significance was calculated using a two-way ANOVA followed by a Sidak's multiple comparison *post hoc* test. *p ≤ 0.05, **p ≤ 0.01, ***p ≤ 0.001.

Agonist induced platelet activation is vital for normal haemostasis and thrombosis, with abnormal responses resulting in a bleeding diathesis. It is possible that incubation of platelets with QDs could inhibit platelet activation in response to agonist stimulation. We have previously found that incubation of washed platelets with 100 nM QD-Pen or QD-LA decreased their aggregation response to thrombin.^17^ Due to this we assessed whether washed platelets were still able to aggregate in response to thrombin after incubation with PEGylated QDs. Washed platelets were incubated with QDs for 10 min before stimulation with 0.1 U mL^−1^ thrombin (Fig. 4). As we have previously observed, 30 and 100 nM QD-LA caused platelet activation which resulted in decreased thrombin responses. However, PEGylation of QD-LA and QD-Pen significantly recovered normal activation to thrombin. The same trend was observed when collagen was used to activate platelets (Fig. S23, ESI†).

**Fig. 5:**
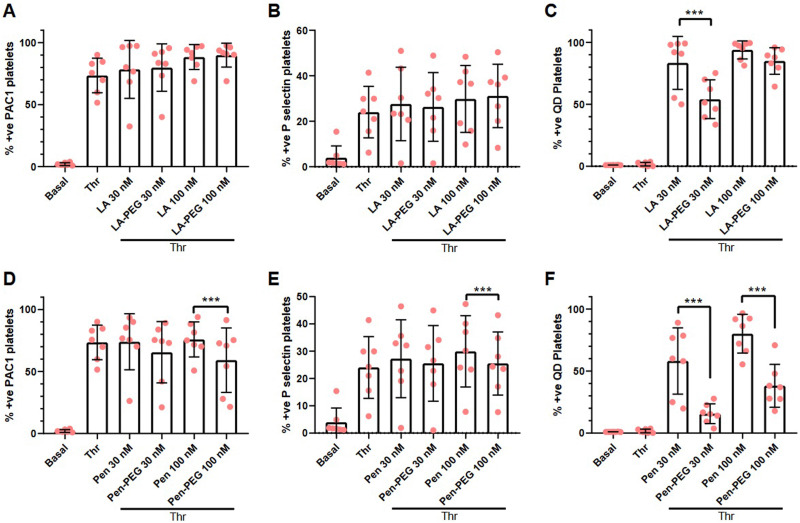
Platelets incubated with QDs are still able to express activation markers in response to thrombin. Washed platelets at 1 × 10^7^ mL^−1^ were incubated with monoclonal antibodies against P-selectin, CD42b, and PAC1 for 5 min before the addition of either QD-LA, QD-LA-PEG, QD-Pen, or QD-Pen-PEG (at either 30 nM or 100 nM) for 20 min. 0.1 U mL^−1^ thrombin (Thr) was used to stimulate platelets. Platelets were fixed after 10 min with 0.45% PFA before being analysed by flow cytometry. The number of platelets positive for (A) and (D) PAC1, (B) and (E) P-selectin and (C) and (F) QDs are shown. Data is shown as mean ± SD. *n* = 7. Statistical significance was calculated using a two-way ANOVA followed by a Sidak's multiple comparison *post hoc* test. *p ≤ 0.05, **p ≤ 0.01, ***p ≤ 0.001.

In agreement with the above data, flow cytometry analysis showed that the addition of thrombin had no effect on integrin activation or P-selectin exposure in samples treated with QDs that induced aggregation by themselves, as platelets were already fully activated (Fig. S24, ESI†), whilst integrin activation and P-selectin exposure was able to be induced effectively in the presence of PEGylated QDs (Fig. 5). In contrast, levels of QD-Pen bound to platelets was reduced upon PEGylation and this was also the case for QD-LA-PEG at 30 nM, indicating that increased QD-platelet interaction could underlie increased activation.

### Platelet spreading is maintained in the presence of PEGylated QDs

To further examine how platelet activation is affected by QDs, platelet spreading assays were undertaken. Platelets were first incubated with relevant concentrations of QD-Pen, QD-Pen-PEG, QD-LA, or QD-LA-PEG and spread on either 100 μg mL^−1^ collagen or 100 μg mL^−1^ fibrinogen coated coverslips for 25 min.

**Fig. 6:**
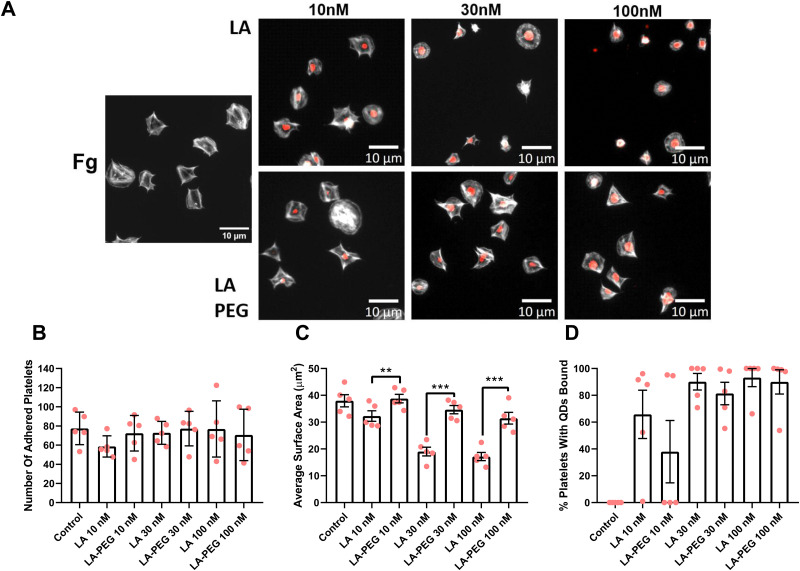
Platelet spreading is maintained in the presence of PEGylated LA QDs. Washed platelets at 2 × 10^7^ mL^−1^ were spread on 100 μg mL^−1^ fibrinogen (Fg) in the absence, or presence of either QD-LA or QD-LA-PEG at 10 nM, 30 nM or 100 nM for 25 min before permeabilizing, fixing and staining with FITC-Phalloidin. Coverslips were mounted and visualized by fluorescence microscopy using an NA 1.4 oil x63 objective. (A) Representative images with scale bars showing 10 μm. Graphs show (B) number of adhered platelets, (C) average surface area of platelets (μm^2^), and (D) percentage of platelets with QDs bound. Data is shown as mean ± SD. *n* = 5. Statistical significance was calculated using a two-way ANOVA followed by Sidak's multiple comparison *post hoc* test. *p ≤ 0.05, **p ≤ 0.01, ***p ≤ 0.001.

**Fig. 7:**
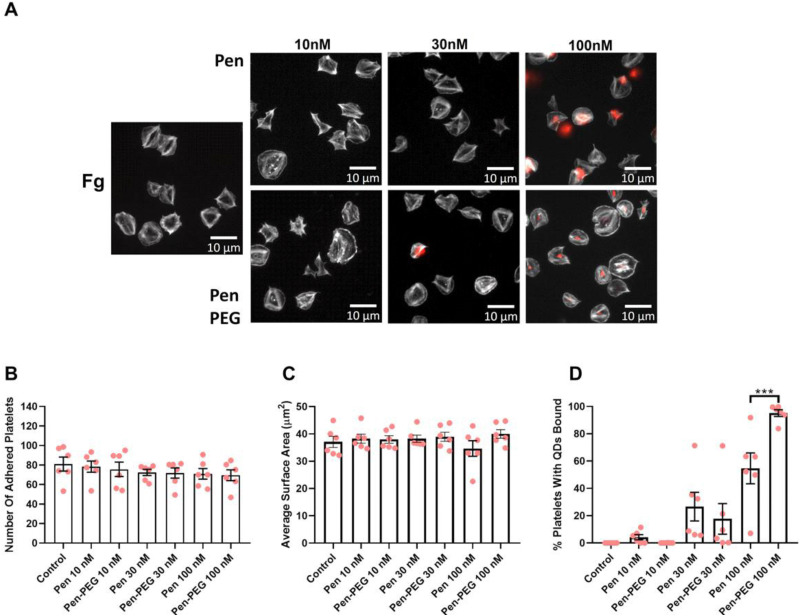
Platelet spreading is maintained in the presence of PEGylated Pen QDs. Washed platelets at 2 × 10^7^ mL^−1^ were spread on 100 μg mL^−1^ fibrinogen (Fg) in the absence, or presence of either QD-Pen or QD-Pen-PEG at 10, 30 or 100 nM for 25 min before permeabilizing, fixing and staining with FITC-Phalloidin. Coverslips were mounted and visualized by fluorescence microscopy using an NA 1.4 oil x63 objective. (A) Representative images with scale bars showing 10 μm. Graphs show (B) number of adhered platelets, (C) average surface area of platelets (μm^2^), and (D) percentage of platelets with QDs bound. Data is shown as mean ± SD. *n* = 6. Statistical significance was calculated using a two-way ANOVA followed by Sidak's multiple comparison *post hoc* test. *p ≤ 0.05, **p ≤ 0.01, ***p ≤ 0.001.

As shown previously,^17^ QD-LA decreased platelet surface area in a dose-dependent manner, whilst the number of adhered platelets remained the same (Fig. 6). However, PEGylation of QD-LA restored the surface area of platelets, with 100 nM QD-LA-PEG having no significant difference in surface area compared to the control. Interestingly, QD-LA was observed to bind to platelets at all concentrations and this was not remedied by PEGylation. QD-Pen and QD-Pen-PEG had no significant changes in platelet adherence and surface area when compared to the control (Fig. 7). QD binding to platelets was observed at 100 nM QD-Pen, which was further increased by PEGylation.

PEGylation of QD-LA, and QD-Pen induced a similar re-establishment of platelet spreading on collagen (Fig. S25 and S26, ESI†). Interestingly, although binding of QD-LA and QD-LA-PEG was also of similar levels to the results seen with fibrinogen spreading, QD-Pen-PEG did not bind platelets at any of the concentrations tested on collagen.

### Effect of QD PEGylation on thrombus formation

As all previous assays were completed in washed platelet preparations, it was critical to understand platelet QD interactions within a physiological setting. Therefore, an *in vitro* thrombus stability assay was performed in the absence of a functional coagulation system. Anti-coagulated whole blood was incubated with either QD-Pen, QD-Pen-PEG, QD-LA, or QD-LA-PEG before flowing over a microfluidic chip coated with collagen at an arterial shear rate of 1000 s^−1^.

**Fig. 8:**
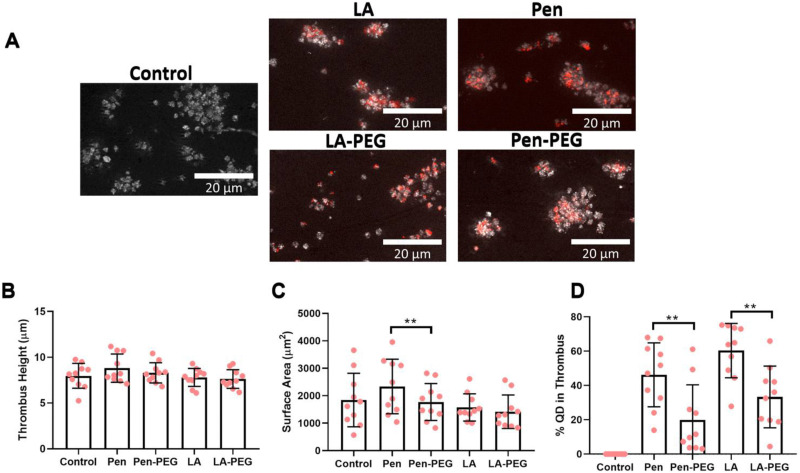
Effect of PEGylated QDs on thrombus formation. Whole blood was stained with DiOC_6_, incubated with 100 nM of relevant QD for 20 min, and then flowed at 1000 s^−1^ arterial shear for 4 min over a microfluidic biochip pre-coated with 100 μg mL^−1^ collagen. The chip was fixed, then imaged with a fluorescence microscope using an NA 1.4 oil x63 objective. Representative images of thrombus formation with scale bars showing 20 μm shown in (A). Graphs show (B) thrombus height (μm), (C) thrombus surface area (μm^2^), and (D) percentage of QDs in platelets. Data is shown as mean ± SD. *n* = 10. Statistical significance was calculated using a one-way ANOVA followed by Tukey's multiple comparison *post hoc* test. *p ≤ 0.05, **p ≤ 0.01, ***p ≤ 0.001.

The thrombus height was unaffected by any of the QDs when compared to the control (Fig. 8). However, surface area was more variable. No group was statistically different to the control, but there was a difference between QD-Pen and QD-Pen-PEG. Importantly, PEGylation of both QD-LA and QD-Pen significantly reduced levels of QDs in the platelet rich thrombus, indicating these QDs interacted less with the thrombus itself.

## Discussion

The use of QDs as an imaging tool has seen rapid development in recent years, with multiple types of QDs developed and investigated in various cell types.^22–25^ However, little investigation has been completed into how these QDs affect platelet function. Platelets are vital cells which are integral to haemostasis. These cells can also endocytose small particles or bacteria and therefore could potentially engulf QDs.^26–28^ Previous work investigating platelet responses to QDs used Cd QDs,^15^ which have now been shown to have biocompatibility and toxicity issues.^29^ Therefore, InP/ZnS QDs were developed, and have been shown to be more biocompatible.^30–33^ Crucially we have previously shown that these QDs can induce platelet responses, dependent on the surface coating of the QD.^17^ Therefore, as PEGylation of molecules and drugs has previously been used in other QD systems to reduce QD mediated cell responses,^34–38^ we investigated if PEGylation of InP/ZnS QDs would block QD mediated platelet activation. Herein, we show using a range of different platelet assays that PEGylation: (1) markedly prevents QD mediated platelet activation, (2) improves the viable concentration range by which QDs could be used without causing platelet activation, and (3) does not affect thrombus formation in whole blood, and reduces QD-thrombi interaction. This data demonstrates that InP/ZnS QDs have the potential to be developed further as drug delivery and imaging agents if coated in the most appropriate manner.

InP/ZnS QDs were prepared according to literature protocols, and the resulting nanoparticles had physical and optical properties in line with these reports.^17,19^ The QD cores have the expected structure and In : P ratio for the material. The increase in diameter following shelling corresponds to a shell of thickness 0.455 nm; this is less than 1 unit cell thick (α(ZnS) = 5.345 Å) and is in agreement with the increase in Zn : In ratio observed by EDX (calculated ratio = 85 : 15, observed = 88 : 12 ± 1.5, Table S1, ESI†). The increased phosphorous content following shelling is likely due to the use of trioctylphosphine as a reagent during the shelling process; this can act as a coordinating ligand.^17^

The QDs were synthesised with hydrophobic ligands in organic media and therefore, for biological application, transfer to aqueous media is essential. Phase transfer was undertaken using two ligands – Pen and LA – under reducing basic conditions. This results in the cleavage of any disulfide bonds and the attachment of small hydrophilic thiols to the QD surface allowing for dispersion in aqueous media. These two ligands were selected based on our previous studies as examples of systems with minimal and significant interactions with platelets respectively.^17^ The decrease in nanoparticle diameter observed following phase transfer may be due to removal of some Zn from the surface of the shell, evidenced by the reduction in Zn : In ratio (Table S1, ESI†). A distinct shift in Zn : S ratio is also observed, supporting both the removal of some Zn atoms and the addition of thiol ligands. This is less pronounced in QD-LA than in QD-Pen; less Zn is removed (evidenced through both Zn : In and Zn : S ratios, Table S1, ESI†). Alongside this the organic fraction of each sample, as determined by TGA, corresponds to 794 and 438 ligands per QD for QD-Pen and QD-LA respectively. The differences with both the removal of Zinc and organic fractions may be due in part to the bidentate nature of the reduced LA in comparison to the monodentate Pen ligand.

The decrease in zeta potential of QD-Pen when compared to QD-LA is likely due to the amphoteric nature of the Pen ligand, resulting in a lower overall charge per ligand. This difference in charge may be the cause of the difference in *D*_H_, or this might be due to the increased number of ligands on the surface of QD-Pen promoting an increased amount of water molecules to be associated with the nanoparticles. The differences in emission wavelength and *φ* upon phase transfer and between surface ligands was previously attributed to a change of the dielectric environment and surface charge.^21^ The longer fluorescent lifetime of QD-LA compared to QD-Pen may be indicative of a reduction in non-radiative decay pathways for this system, this may be the cause of the increased quantum yield. This would presumably be due to a more complete shelling in QD-LA than in QD-Pen, as a result of the removal of less Zn during the phase transfer reaction.

PEGylation was investigated as a route to reduce QD mediated platelet effects. PEGylation is a technique that has been used clinically to improve the biocompatibility of drug preparations. It can have multiple effects, increased stability, changing the chemical and physical properties, and also reducing the immunogenicity of the drug compound.^18^ In this study we used PEGylation to prevent unwanted platelet activation by QD preparations that we had previously identified as problematic.^17^ PEGylation of the QDs had the desired effect, decreasing the extent of QD-associated platelet aggregation, integrin activation and P-selectin exposure for both QD-Pen and QD-LA. This reduction in QD driven platelet activation also meant that platelets were then able to respond to physiological agonists as expected. This data clearly demonstrates that PEGylation offers a profound benefit to QD preparations with regards to the reduction of platelet activation. This decrease in QD mediated platelet activation in the presence of PEG is similar to that seen with gold nanoparticles, where PEGylation offered increased biocompatibility to platelet aggregation,^37^ whilst Bakhaidar *et al.* also showed no effect of PEG-PLGA nanoparticles on platelet aggregation and P-selectin exposure.^39^

Although the effect of PEGylation was beneficial, there were assay specific effects. In the platelet spreading assay, PEGylation of QDs restored the ability to platelets to spread effectively in comparison to unPEGylated QDs. However, the interaction of QDs with platelets was surface specific. Platelets spread on collagen did not interact with PEGylated QDs, whilst platelets spread on fibrinogen still had significant QD-platelet interactions. The matrix protein dependent nature of QD-platelet interaction is of interest as collagen is present in the subendothelial matrix, whilst fibrinogen, which is then changed into fibrin, is present within a thrombus itself.^40^ However, one significant caveat with the spreading assay is that there is no shear force present, and therefore QDs can interact more easily with the platelets spread on the matrix proteins. This is demonstrated through comparison with the results from the flow cytometry and *in vitro* flow assays. Within flow cytometry, where the platelets and QDs are in suspension, PEGylation of both QD-LA and QD-Pen decreased QD binding to platelets, even under stimulated conditions. The *in vitro* flow assay includes a shear force of 1000 s^−1^, similar to that seen within arteries.^41^ Again, the amount of QD in the thrombus was significantly reduced for both QD-LA-PEG and QD-Pen-PEG. This highlights that the specific conditions of the assay could in part be responsible for the effectiveness of the QD platelet interactions, and therefore potentially the effect of the QD on platelet activation.

Additionally, within the spreading assay, the QDs that did interact were present in or on the centre of the platelet. Although it is not possible to distinctly identify if the QDs are within or on the outside of the platelet due to resolution limitations, another study has found that when QDs were interacting with platelets, platelet organelles were centralised within the platelet, with degranulation having occurred.^15^ This was suggested to be due to QD interaction with GPIIb/IIIa. This is similar to the proposed mechanism of action for platelets binding to bacteria, resulting in bacteria localisation to the centre of platelets close to organelles, in which GPIIb/IIa has a large role,^42^ which allows for spatial regulation of granule secretion,^43^ potentially towards the QD. Further research is needed to fully understand how and why QDs interact, and what the specific effect on the platelet is when that interaction occurs.

Previous studies have assessed the impact of zeta potential on platelet activation. CdSe/ZnS functionalised with carboxyl caused thrombosis in pulmonary vasculature. The amine-functionalised QDs with a less negative zeta potential did not cause thrombosis to the same extent. The authors hypothesised that the QDs were affecting coagulation more than platelets themselves, and it was this effect on coagulation which had a knock-on effect on platelets.^16^ Interestingly, we observed the zeta potential of QD-LA to be more negative than that of QD-Pen. PEGylation of both QDs resulted in less negative zeta potentials, though QD-LA-PEG was more negative than that of QD-Pen. This corresponds to the levels of platelet activation the QDs elicit. Hence, zeta potential could act as a determinant for platelet activation. As washed platelets do not contain coagulation factors, our results would suggest that whilst QDs in other systems could be utilising coagulation factors to further increase platelet activation, platelets alone are also strongly activated by QDs. It would be interesting to further look at the effects of PEGylated QDs on coagulation factors.

Size of the QD has also been linked to activation of platelets, with QDs of smaller size having been observed in Samuel *et al.* to cause higher levels of platelet activation.^15^ The mechanism for the increased activation effects of smaller QDs is thought to be due to a greater surface-to-volume area which would allow for more QD interactions with platelets. We have previously shown QD-LA and QD-Pen to have a diameter of 6.0 and 4.1 nm respectively, and yet the smaller QDs induce less platelet activation.^17^ Here again, PEGylated QDs were either the same size (QD-Pen and QD-Pen-PEG) or the QD-LA PEG was smaller than the QD-LA itself. Therefore, size is likely to be less relevant that charge and surface coating of the QDs.

To conclude, the above studies have demonstrated that PEGylation of QDs is a viable option to reduce QD mediated platelet activation. We have shown PEGylated permutations of QDs that initially cause significant activation, cause markedly less platelet activation upon PEGylation. This in turn allowed the platelet to retain the ability to respond to platelet agonists. Furthermore, the PEGylated QDs showed reduced interaction with basal and activated platelets, both in washed platelets and crucially in whole blood, making them preferable for further development for QD mediated therapies or imaging agents for thrombus management *in vivo*.

## Author contributions

LNA, ZB, and SVLL designed, carried out, analysed the biology data, and wrote the manuscript. TWP, JG, LDT, SH-S, NTKT, ZA, NH, TC, J-SGB, designed, carried out, analysed the chemistry data, and wrote the manuscript. DA, JS, KM wrote and edited the manuscript. GJS and SDJC designed the experiments, wrote, and edited the manuscript.

## Data availability

The data within this study is included in either the main article or ESI† figures.

## Conflicts of interest

The authors have no conflicts of interest.

## Supplementary Material

TB-013-D4TB01334D-s001
