# Supplementary material for: PEGylation of indium phosphide quantum dots prevents quantum dot mediated platelet activation
Source: J Mater Chem B. 2024 Dec 5;13(3):1052–63. doi: 10.1039/d4tb01334d (PMC11619005; doi:10.1039/d4tb01334d)
Supplement: TB-013-D4TB01334D-s001 [file TB-013-D4TB01334D-s001.pdf]

Supplementary Information (SI) for Journal of Materials Chemistry B.
This journal is © The Royal Society of Chemistry 2024

Experimental methods

Regents

Indium chloride ($InCl_3$, 99.999%, Thermo Scientific), zinc chloride ($ZnCl_2$, 99.999%, Sigma-Aldrich), oleylamine (technical grade, 70%, Sigma-Aldrich/Merck), tris(diethylamino)phosphine ($P(NEt_2)_3$, 97%, Alfa Aesar), sulfur (99.98%, Sigma-Aldrich), trioctylphosphine (TOP, technical grade, 90%, Thermo Scientific), zinc searate (technical grade, Sigma-Aldrich), 1-octadecene (technical grade, 90%, Aldrich), chloroform (99.0-99.4%, Sigma-Aldrich/Merck), ethanol (>99.8%, Sigma-Aldrich/Merck), hexane (>95%, Sigma-Aldrich/Merck), water (obtained from a Veolia Purelab Chorus system, with a resistivity value of 18 MΩ corrected at 25 ^{o}C), tetramethylammonium hydroxide (TMAOH, 25% weight in H_2O, Sigma Aldrich), tris(2-carboxyethyl)phosphine hydrochloride (TCEP.HCl, Fluorochem), penicillamine (pen) (Fluorochem), lipoic acid (LA) (Fluorochem), and O-[2-(3-Mercaptopropionylamino)ethyl]-O′-methylpolyethylene glycol (M_w = 5,000 gmol^{-1}, Sigma-Aldrich) were purchased from commercial sources. All chemicals were used as received, without further purification. During synthesis samples were centrifuged using a Jouan CR312 centrifuge.

Chrono-Par collagen suspension (Chrono-Log Corporation, Havertown, USA), fibrinogen (Enzyme Research, Swansea, UK), ProLong diamond antifade mountant and paraformaldehyde (ThermoFisher Scientific, UK). Fluorescein Isothiocyanate (FITC) phalloidin, PPACK (D-Phe-Pro-Arg-chloromethylketone), $DIOC_6$ (3,3′ - dihexyloxacarbocyanine iodide) (Enzo Life Sciences, Farmingdale, New York, USA). Vena8 Endothelial+ Microfluidic Biochip (Celix, Dublin, Ireland). Phosphate buffered solution (PBS) tablets (Gibco, Paisley, Scotland). All other chemicals were from Sigma Ltd (Poole, UK) unless otherwise stated.

Physical and Optical Characterisation

Absorption measurements were obtained at 25 °C using a PerkinElmer Lambda 365 spectrophotometer with 10 mm quartz cuvettes. Fluorescence measurements were recorded using an Edinburgh Instruments FS5 spectrofluorometer with 10 mm quartz cuvettes at 25 °C. Quantum yields were measured using an integrating sphere module. Lifetime data was fitted using Fluoracle 2.15.2 with an exponential tail-fit with 4 components using the equation:

$$R(t) = B_1 e^{\left(-t/\tau_1\right)} + B_2 e^{\left(-t/\tau_2\right)} + B_3 e^{\left(-t/\tau_3\right)} + B_4 e^{\left(-t/\tau_4\right)}$$

Fourier transform infrared (FT-IR) spectra were collected using a PerkinElmer Spectrum 100 FT-IR Spectrometer using an ATR attachment. Samples were dried onto the crystal prior to analysis.

Hydrodynamic diameter and zeta potential were determined using a Malvern Panalytical Zetasizer Nano-ZS. Dynamic light scattering measurements were performed in plastic 10 mm cuvettes at 25 °C. Zeta potential measurements were performed in folded capillary zeta cells (Malvern Panalytical) at 25 °C.

Powder X-ray diffraction (PXRD) data were recorded on a Panalytical X’PERT PRO MPD X-ray diffractometer using Cu Kα radiation (λ = 1.540 Å) operated at 45 kV and 40 mA with a scan range from 20° to 90° and a scan step of 0.06°.

Transmission electron microscopy (TEM) and energy dispersive X-ray (EDX) spectroscopy were performed as follows. QDs were drop-cast onto a copper grid with a graphene oxide film on lacey carbon, for optimal image contrast. The TEM was carried out using an FEI Titan^3 Themis 3000 operating at 300 kV, with the FEI Super-X 4-detector EDX system for elemental analysis. The resulting TEM

images were analysed using Gatan Digital Micrograph, within which sizing was performed by manually measuring a statistically relevant number of individual particles (~100-150 QDs per sample).

Thermogravimetric analysis (TGA) of the samples was performed using a Mettler Toledo TGA/DSC 1/1100 SF STARe system from 25 to 900 °C. From room temperature, the samples were heated up to 120 °C at a heating rate of 5 °C/bmin, maintained at 120 °C for 30 min and then heated up to 900 °C at 10 °C/ min, all under an argon flow rate of 30 mL/bmin. Approximately 5 mg sample were used for the analysis.

Synthetic Procedures

Synthesis of Core-Shell InP/ZnS QDs

QDs were synthesized according to literature procedures (17, 19),

Phase Transfer

Phase transfer was performed through an adaptation of the procedure reported by Tamang *et al.* (20).

InP/ZnS QDs in hexane (7.4 mL, 300 nmol) were thoroughly purified to remove excess hydrophobic ligands prior to phase transfer. The hexane solution was centrifuged (3600 rpm, 6 min and the supernatant combined with ethanol (3x volume, 22 mL). The suspension was centrifuged (3600 rpm, 6 min) and the supernatant discarded. The pellet was resuspended in chloroform (5 mL) and then combined with ethanol (3x volume, 15 mL). The suspension was centrifuged (3600 rpm, 6 min) and the supernatant discarded. The pellet was resuspended in 5 mL chloroform.

Penicillamine (456 mg, 3 mmol, 10 000 eq) or lipoic acid (632 mg, 3 mmol, 10 000 eq) were combined with TCEP.HCl (439 mg, 1.5 mmol, 5 000 eq). The reagents were suspended in water and the pH adjusted with TMAOH to produce a clear solution with final volume of 8 mL at the appropriate reaction pH (11.0 for penicillamine, 11.9 for lipoic acid).

The QDs in chloroform and the thiol ligands in water were combined and stirred vigorously in the dark for 1 h. The water layer was separated and purified by spin filtration (Amicon Ultra-4 Ultracel – 30 K regenerated cellulose centrifugal filters, Merck Millipore) with a minimum of 5 repeats of 10x dilution, or until the solution was neutral, to remove excess ligands and base. The solutions containing QDs were stored at 4 ^{o}C in the dark.

Ligand Exchange

InP/ZnS QDs in water (2 mL, 88 nmol), HS-PEG^{5k}-OMe (440 mg, 88 µmol, 1000 eq), and TCEP.HCl (76 mg, 264 µmol, 3 000 eq) were combined in water and the pH adjusted using TMAOH to yield a final solution volume of 8 mL and pH of 10. The solution was stirred in the dark overnight. The QDs were purified by spin filtration (Amicon Ultra-4 Ultracel – 30 K regenerated cellulose centrifugal filters, Merck Millipore) with a minimum of 5 repeats of 10x dilution, or until the solution was neutral, to remove excess ligands and base. The solutions containing QDs were stored at 4 ^{o}C in the dark.

Supplemental Figures:

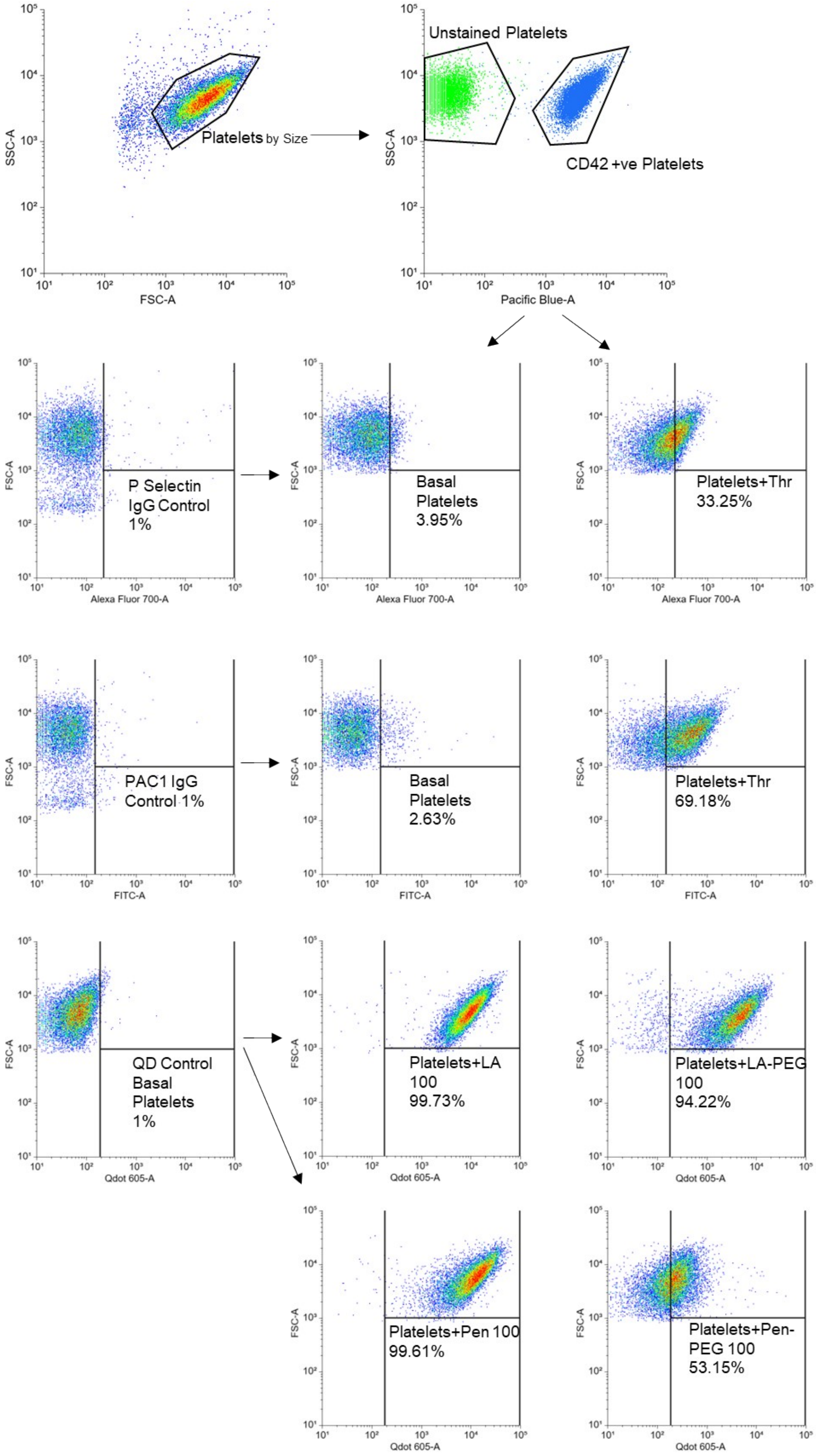

Figure S1 - Experimental settings used for the detection of PAC1, P-selectin, CD42 % positive platelets, and QD binding by flow cytometry. Platelet surface expression of PAC1, P-selectin, and CD42 was analysed in washed platelets on a BD LSR Fortessa cell analyser (BD Biosciences) as described in the Materials and Methods section. (A) Plots depicting the gating strategy for platelets used. Platelets were first gated by size based on physical parameters (size and granularity) on the left. Platelets were next identified as CD42 positive and gated. Right plot shows CD42 positive platelets (blue) and unstained platelets (green) from the previously gated platelets as shown by black arrows. (B) Gating for P-selectin positive platelets. Left plot shows P-selectin isotype control incubated platelets, gated to 1% positive of total population. Middle and right plots show this gate applied to basal and thrombin stimulated platelets respectively. (C) Gating for PAC1 positive platelets. Left plot shows PAC1 isotype control incubated platelets, gated to 1% positive of total population. Middle and right plots show this gate applied to basal and thrombin stimulated platelets respectively. (D) Gating for QD positive platelets. Left plot shows basal gated to 1% positive of total population. Plots show this gate applied to middle top QD-LA 100 nM, top right QD-LA-PEG 100 nM, middle bottom QD-Pen 100 nM, and bottom right QD-Pen-PEG 100 nM.

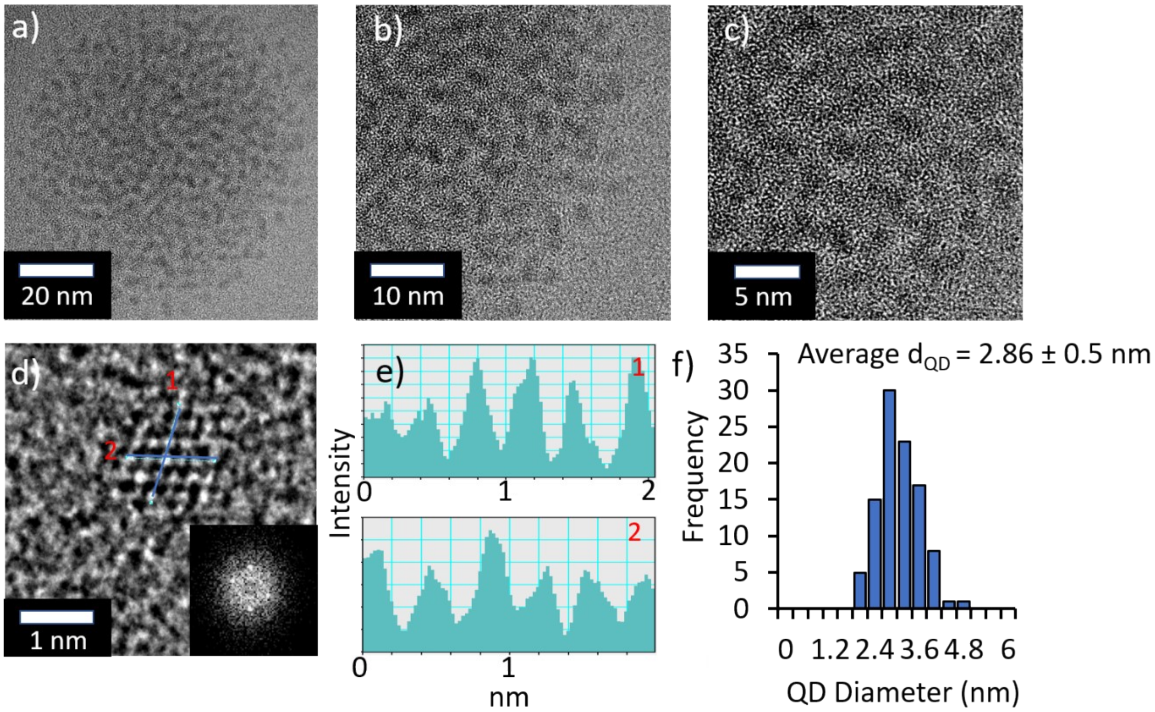

Figure S2: (a-d) HRTEM images of InP cores (electron diffraction inset); (e) corresponding line profiles, and (f) QD size distribution.

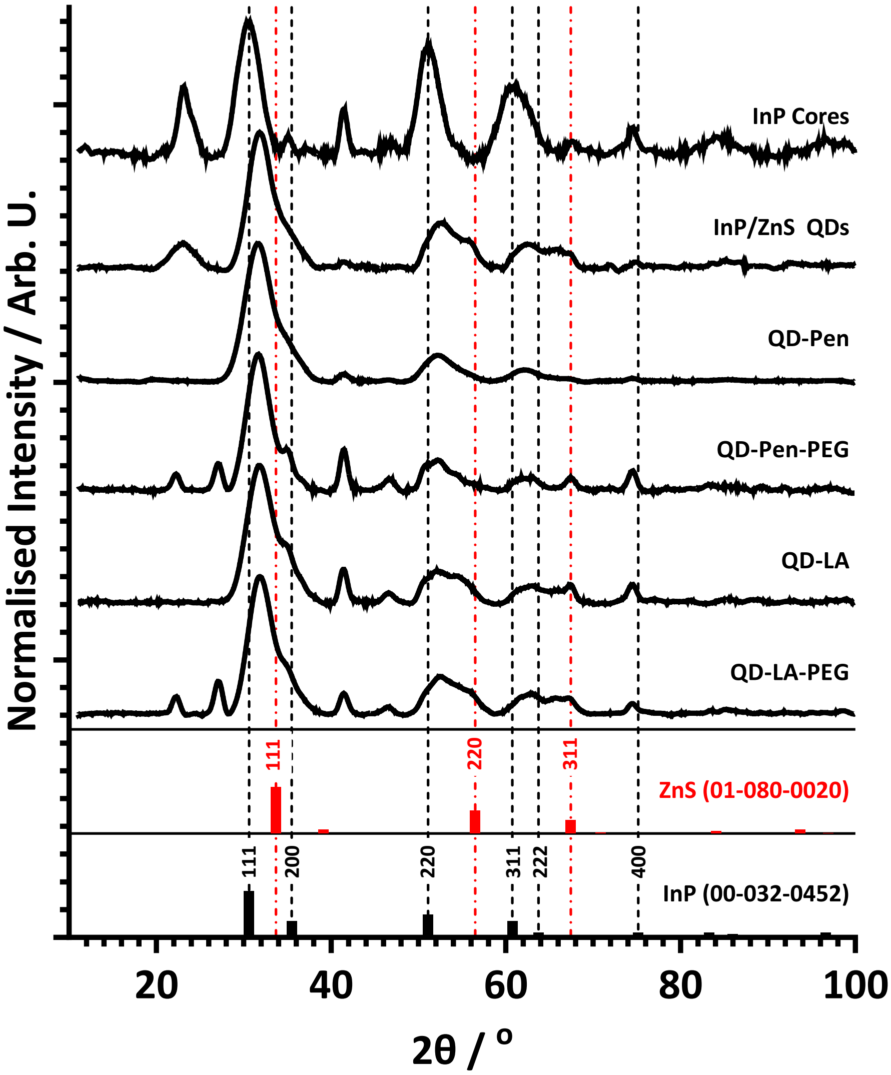

Figure S3: PXRD analysis of QD samples. InP (00-032-0452)[REF-InP] and ZnS (01-080-0020)[Ref – ZnS] references shown as bars, with selected reflections indicated.

Table S1: Physical properties of QD samples

	D_{TEM} / nm	Atomic fraction by EDX / %								
		Zn:In		±	In:P		±	Zn:S		±
**InP**	2.86 ± 0.5	40	60	0.8	49	51	0.7	95	5	0.9
**InP/ZnS**	3.77 ± 0.6	88	12	1.5	17	83	3.2	86	14	3.2
**QD-Pen**	2.63 ± 0.5	61	39	7.7	30	70	8.4	40	60	6.1
**QD-Pen-PEG**	2.82 ± 0.7	70	30	1.7	27	73	7.5	49	51	4.3
**QD-LA**	3.29 ± 0.6	78	22	1.2	41	59	3.7	50	50	2.2
**QD-LA-PEG**	1.99 ± 0.4	81	19	1.7	41	59	3.1	62	38	3.9

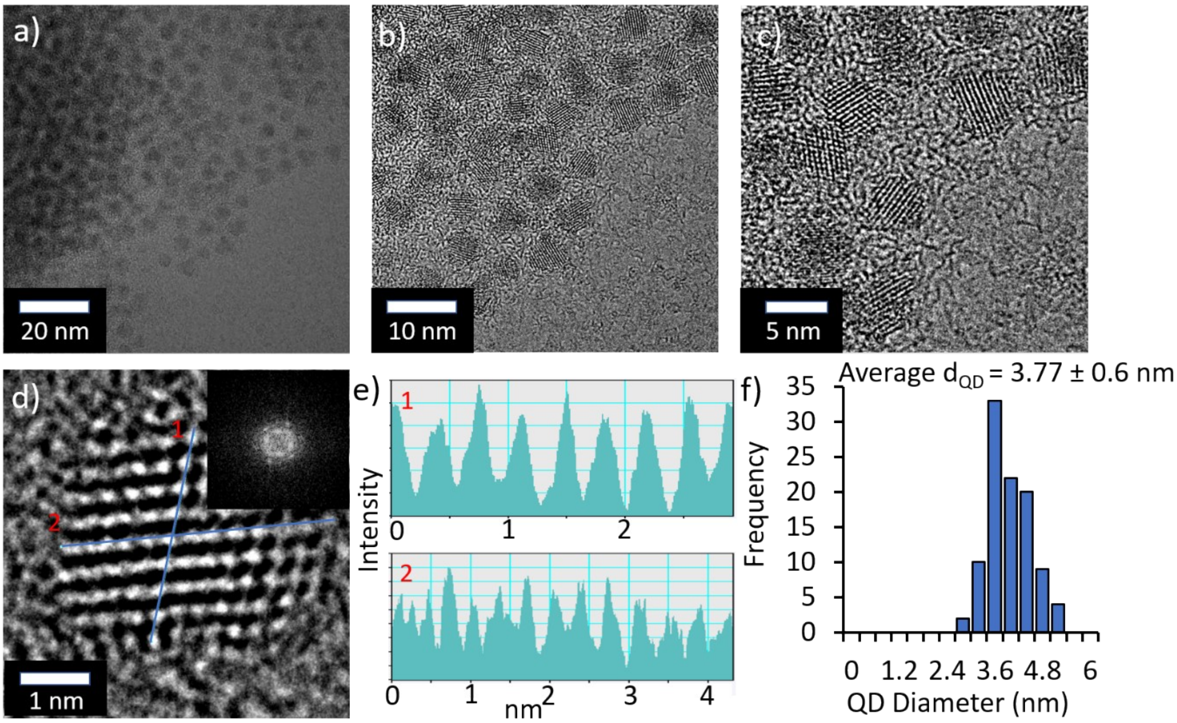

Figure S4: (a-d) HRTEM images of InP/ZnS core/shell QDs (electron diffraction inset); (e) corresponding line profiles, and (f) QD size distribution.

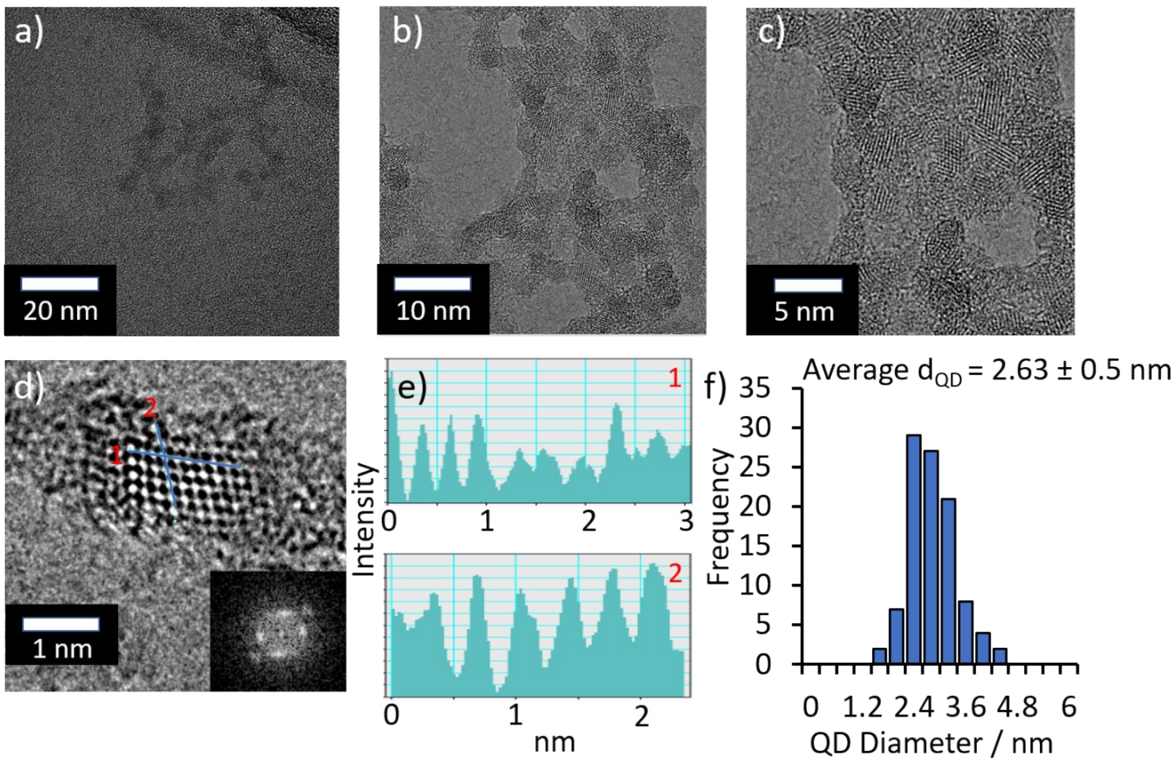

Figure S5: (a-d) HRTEM images of QD-Pen (electron diffraction inset); (e) corresponding line profiles, and (f) QD size distribution.

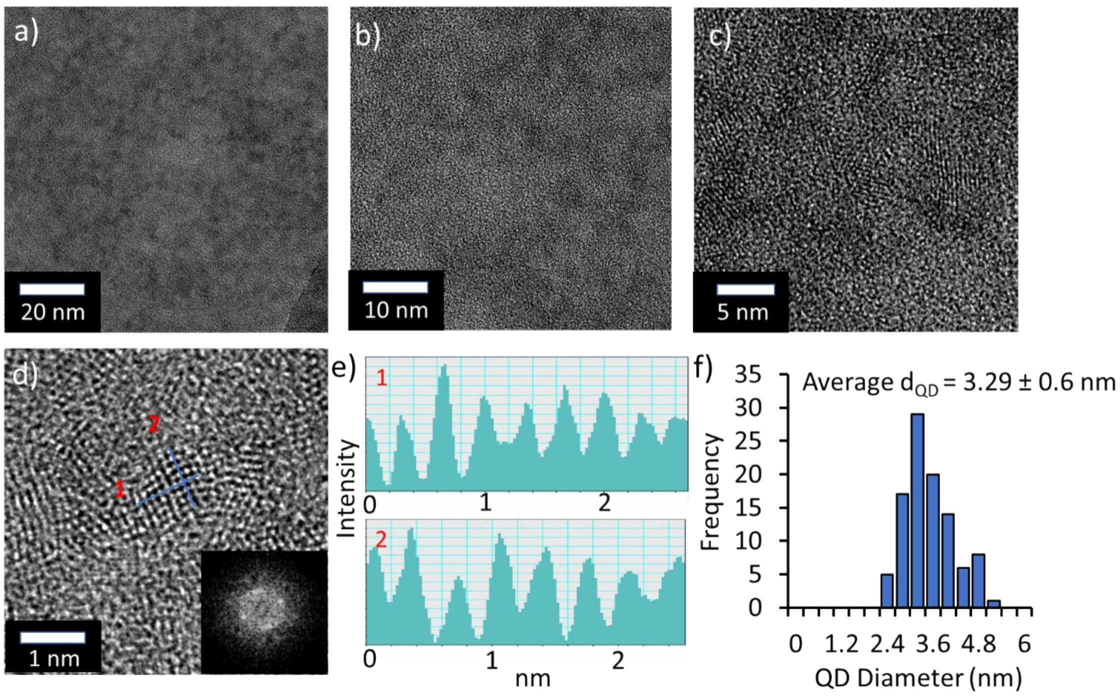

Figure S6: (a-d) HRTEM images of QD-LA (electron diffraction inset); (e) corresponding line profiles, and (f) QD size distribution.

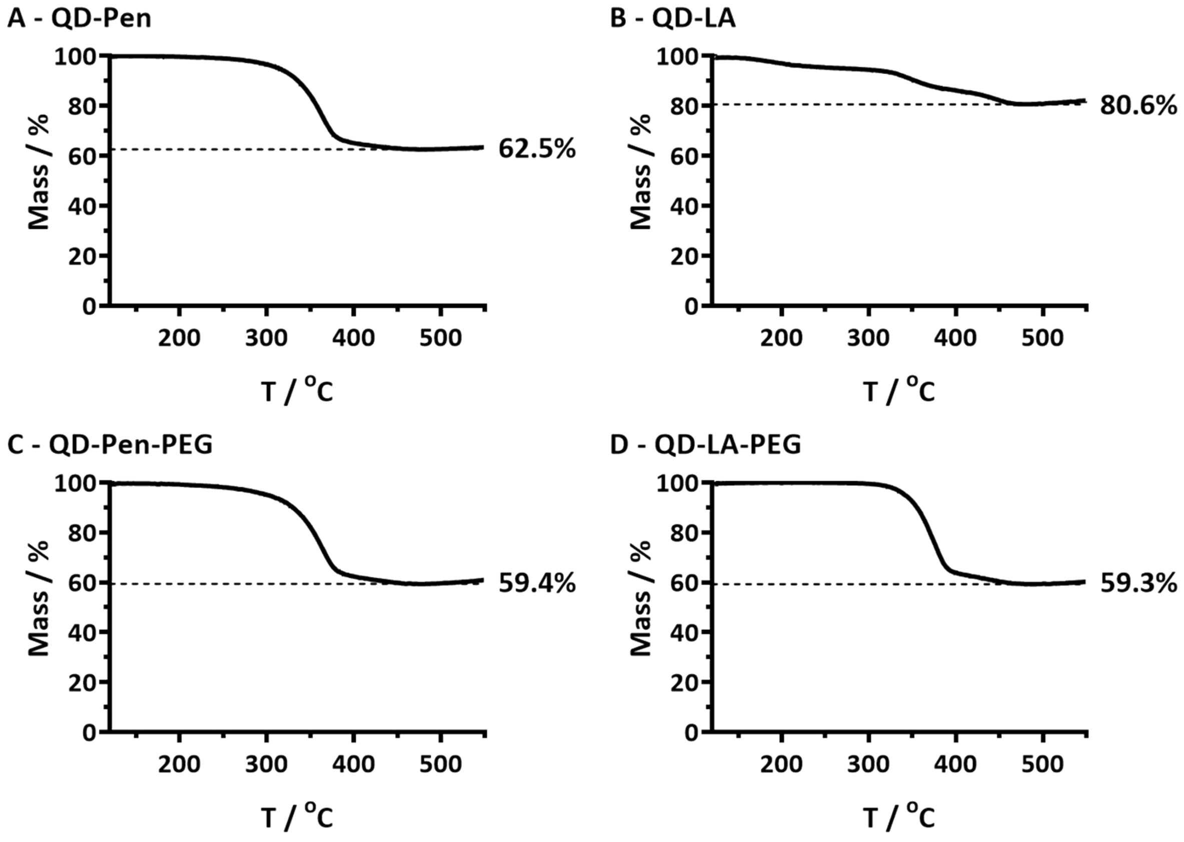

Figure S7: TGA trace obtained for samples, A) QD-Pen, B) QD-LA, C) QD-Pen-PEG, D) QD-LA-PEG. Results are normalised to mass at 120 ^{o}C. Inorganic percentages are indicated.

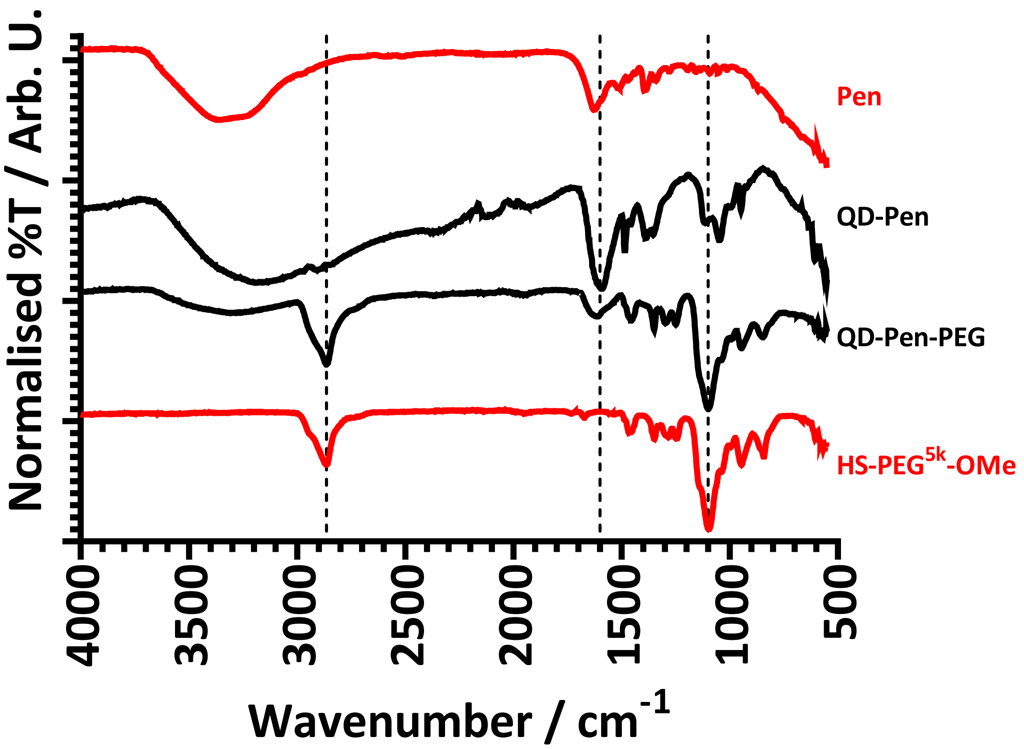

Figure S8: FT-IR spectra of Pen, QD-Pen, QD-Pen-PEG, and HS-PEG^{5k}-OMe, with key peaks highlighted.

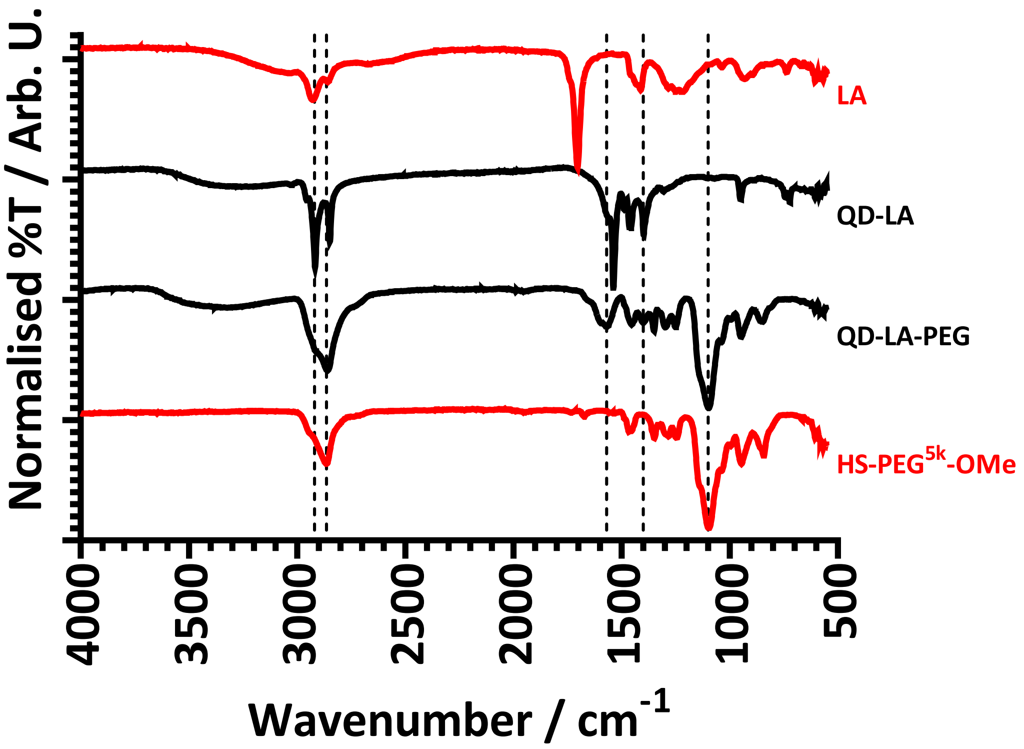

Figure S9: FT-IR spectra of LA, QD-LA, QD-LA-PEG, and HS-PEG^{5k}-OMe, with key peaks highlighted.

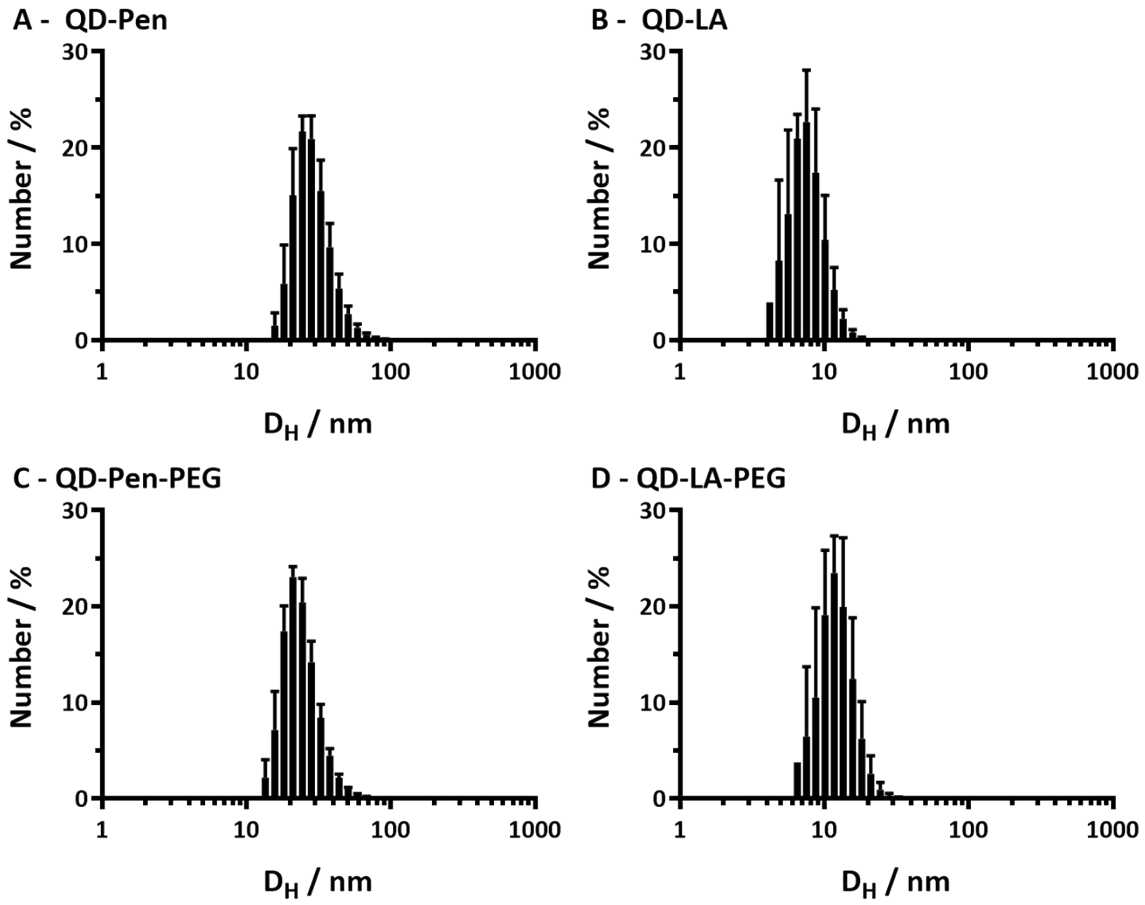

Figure S10: Histograms of DLS of samples. A) QD-Pen, B) QD-LA, C) QD-Pen-PEG, D) QD-LA-PEG. Error bars indicate standard deviation. Measured at 25 ^{o}C in water.

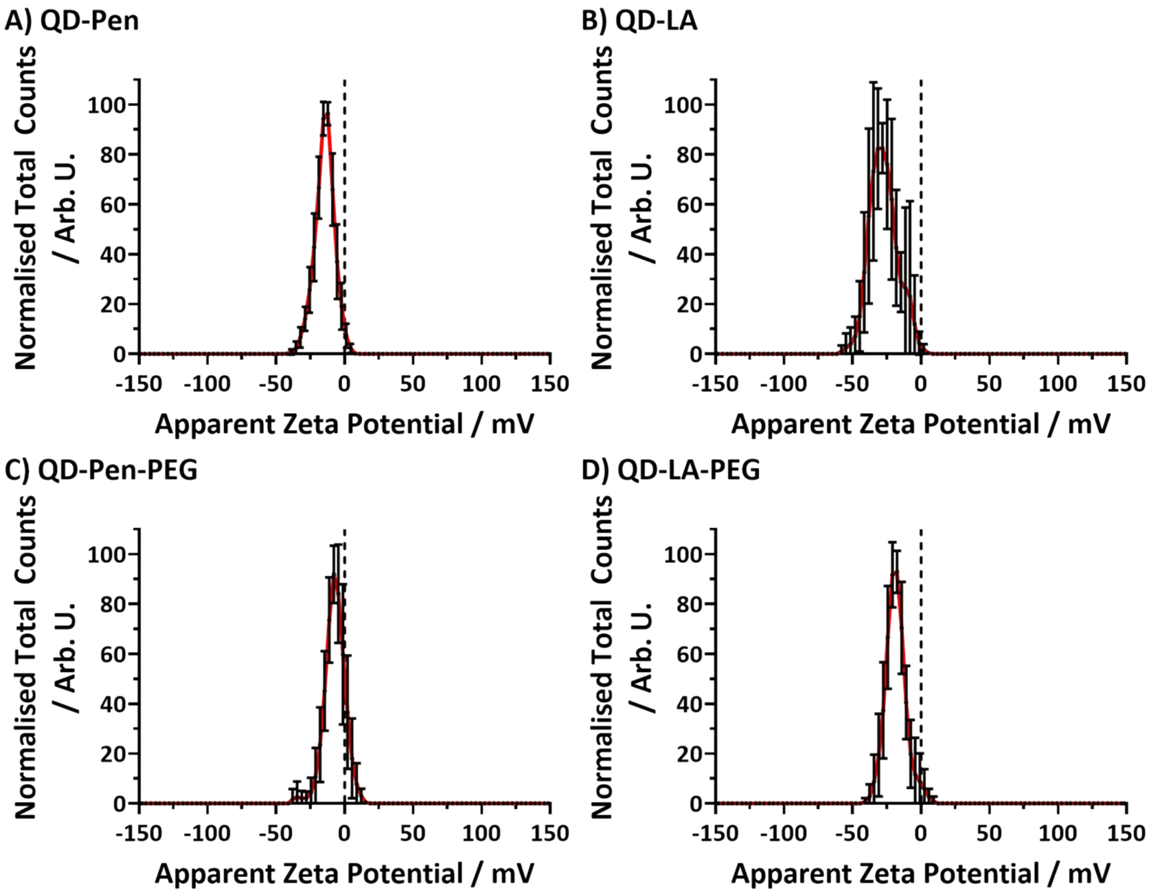

Figure S11: Zeta potentials of samples. A) QD-Pen, B) QD-LA, C) QD-Pen-PEG, D) QD-LA-PEG. Error bars indicate standard deviation. Measured at 25 °C in water.

Table S2: Optical properties of QD samples

	$\lambda_{abs}^{a,b}$ / nm	$\lambda_{max,em}^{b,c}$ / nm	FWHM^{b,c,d} / nm	$\phi^{b,c}$ / %	$\langle\tau\rangle_{amp}^{b,c,e,f}$ / ns
**QD-Pen**	572	611	77	7.5	58.0 ± 3.4
**QD-Pen-PEG**	577	623	78	15.7	62.9 ± 3.5
**QD-LA**	586	633	75	23.6	76.8 ± 5.9
**QD-LA-PEG**	586	632	74	26.7	72.1 ± 3.1

[a] absorbance maxima of excitonic peak. [b] measured in water at 25 °C. [c] λ_{ex} = 405 nm. [d] full-width half-maxima. [e]λ_{em} = 610 nm. [f]relative percentage weighted average of lifetime

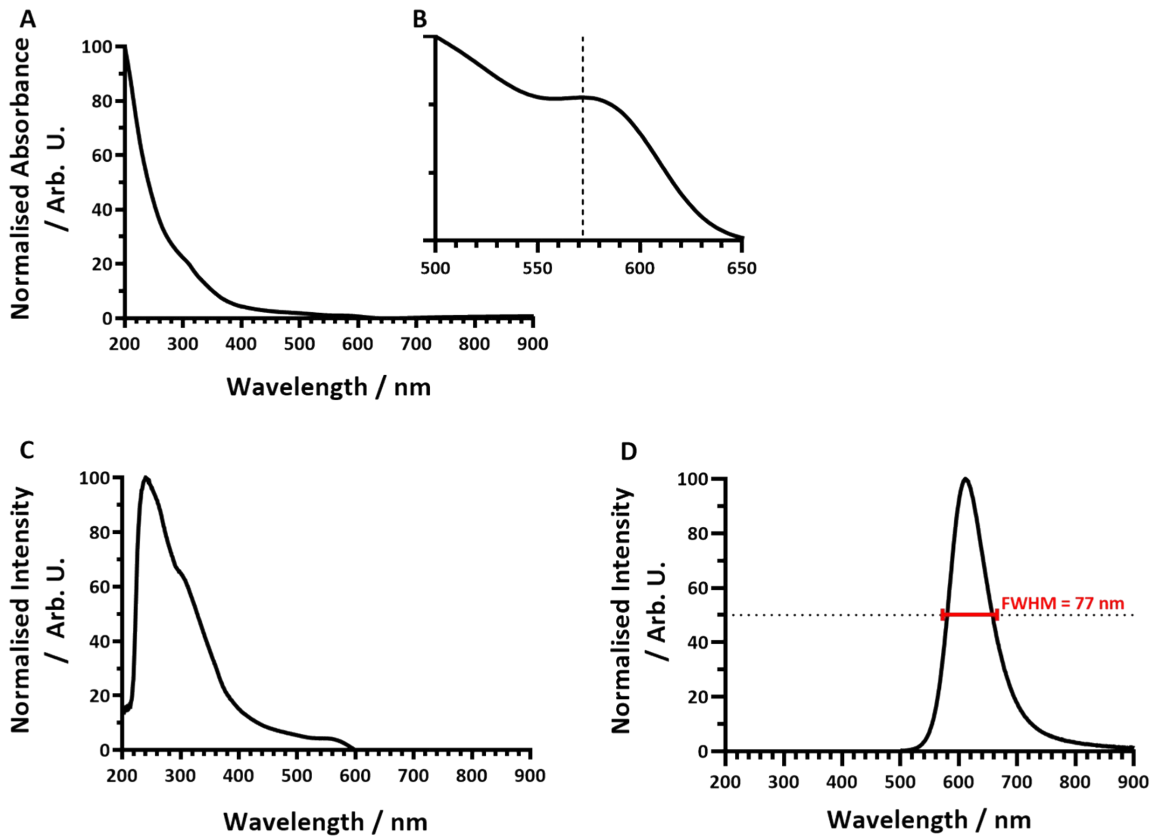

Figure S12 – Optical properties of QD-Pen. A) Absorbance. B) Expanded absorbance around 1st excitonic peak. C) Excitation spectrum, λ_{em} = 610 nm. D) Emission spectrum with FWHM indicated. λ_{ex} = 405 nm. Measurements performed in water at 25 °C.

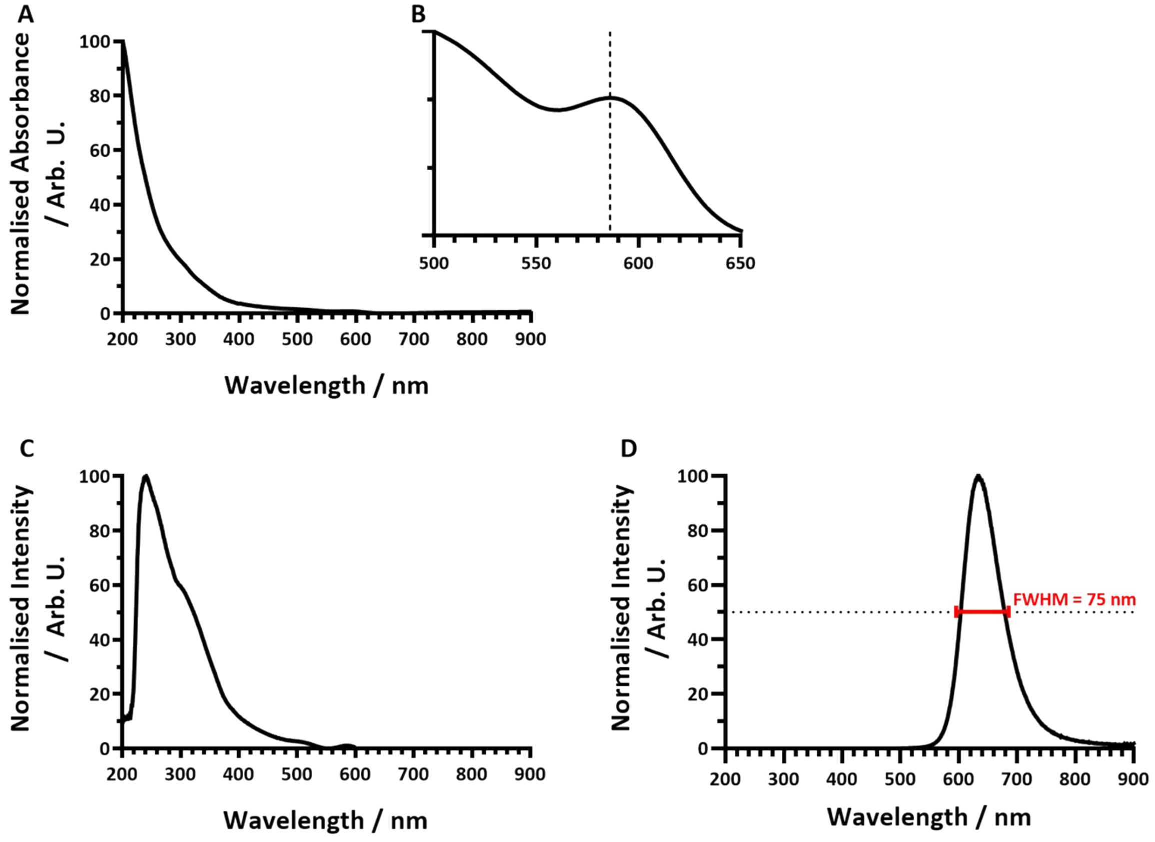

Figure S13 – Optical properties of QD-LA. A) Absorbance. B) Expanded absorbance around 1st excitonic peak. C) Excitation spectrum, λ_{em} = 610 nm. D) Emission spectrum with FWHM indicated. λ_{ex} = 405 nm. Measurements performed in water at 25 °C.

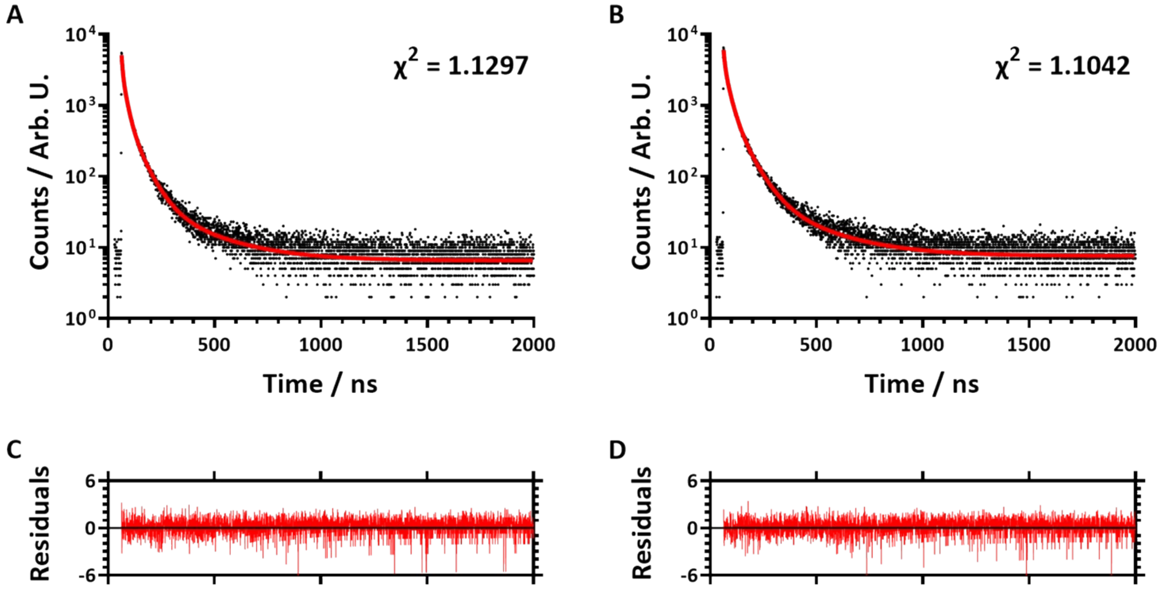

Figure S14 – Fluorescence lifetime decay (black points) and exponential tail fits (red line) for A) QD-Pen, B) QD-Pen-PEG. Residuals of the exponential tail fit are shown for C) QD-PEN, D) QD-Pen-PEG. Measurements performed in water at 25 °C. λ_{ex} = 405 nm, λ_{em} = 610 nm.

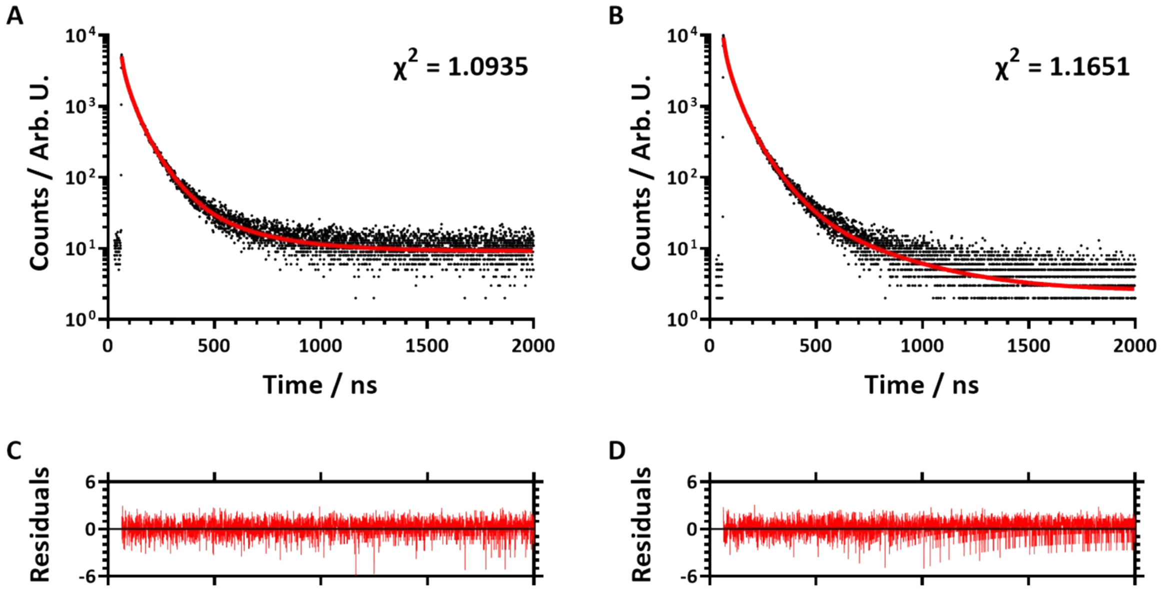

Figure S15 – Fluorescence lifetime decay (black points) and exponential tail fits (red line) for A) QD-LA, B) QD-LA-PEG. Residuals of the exponential tail fit are shown for C) QD-LA, D) QD-LA-PEG. Measurements performed in water at 25 °C. λ_{ex} = 405 nm, λ_{em} = 610 nm.

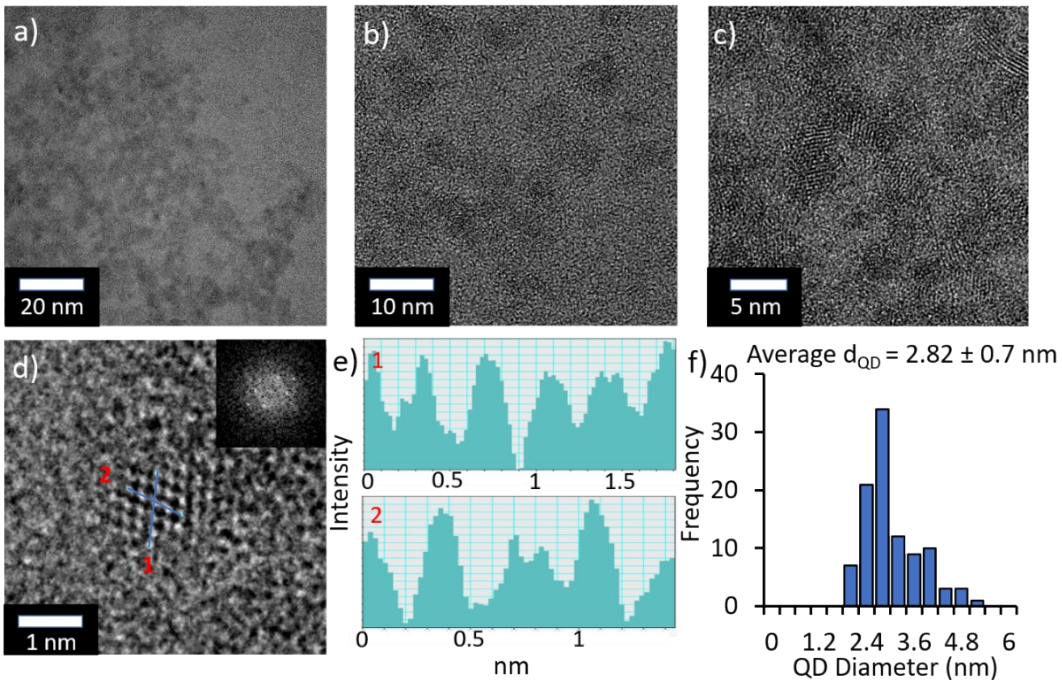

Figure S16: (a-d) HRTEM images of QD-Pen-PEG (electron diffraction inset); (e) corresponding line profiles, and (f) QD size distribution.

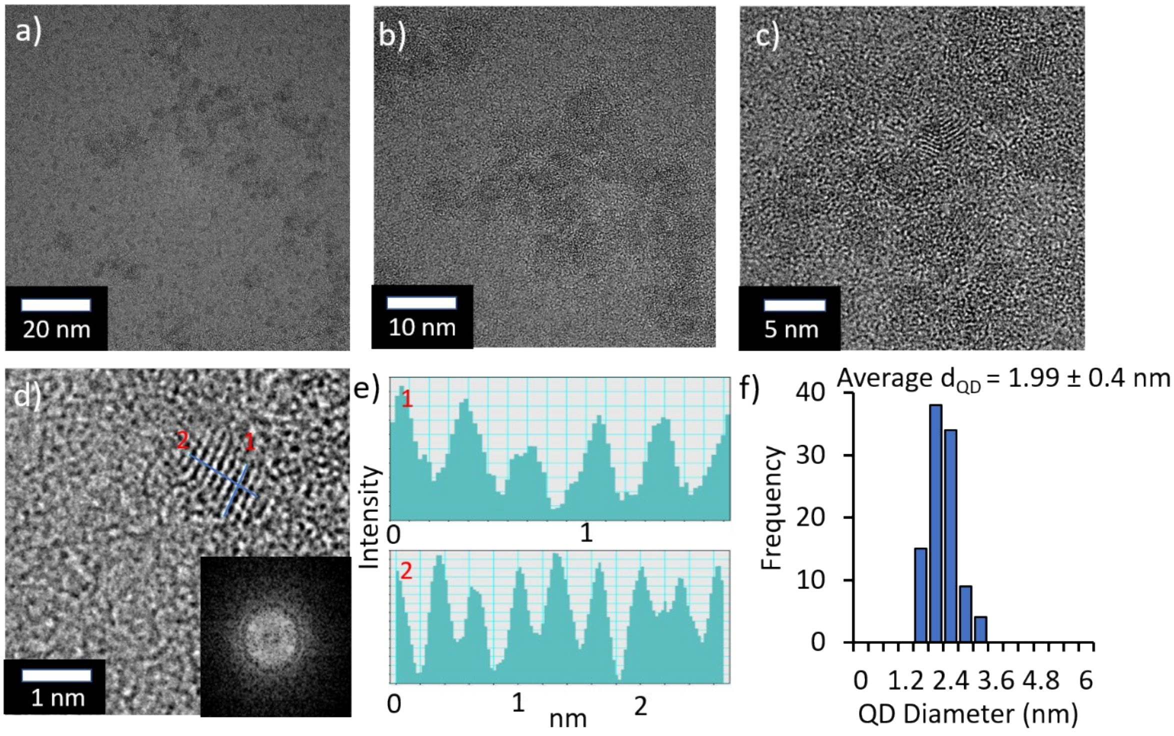

Figure S17: (a-d) HRTEM images of QD-LA-PEG (electron diffraction inset); (e) corresponding line profiles, and (f) QD size distribution.

Table S2: Physical properties of QD samples following phase transfer

	Inorganic fraction / %	D_H / nm	ζ / mV
**QD-Pen**	62.8	29.56 ± 0.90	-14.7 ± 0.31
**QD-Pen-PEG**	60.1	24.63 ± 0.45	-7.40 ± 0.56
**QD-LA**	82.4	7.81 ± 0.48	-27.3 ± 1.39
**QD-LA-PEG**	59.4	12.56 ± 0.72	-18.3 ± 0.57

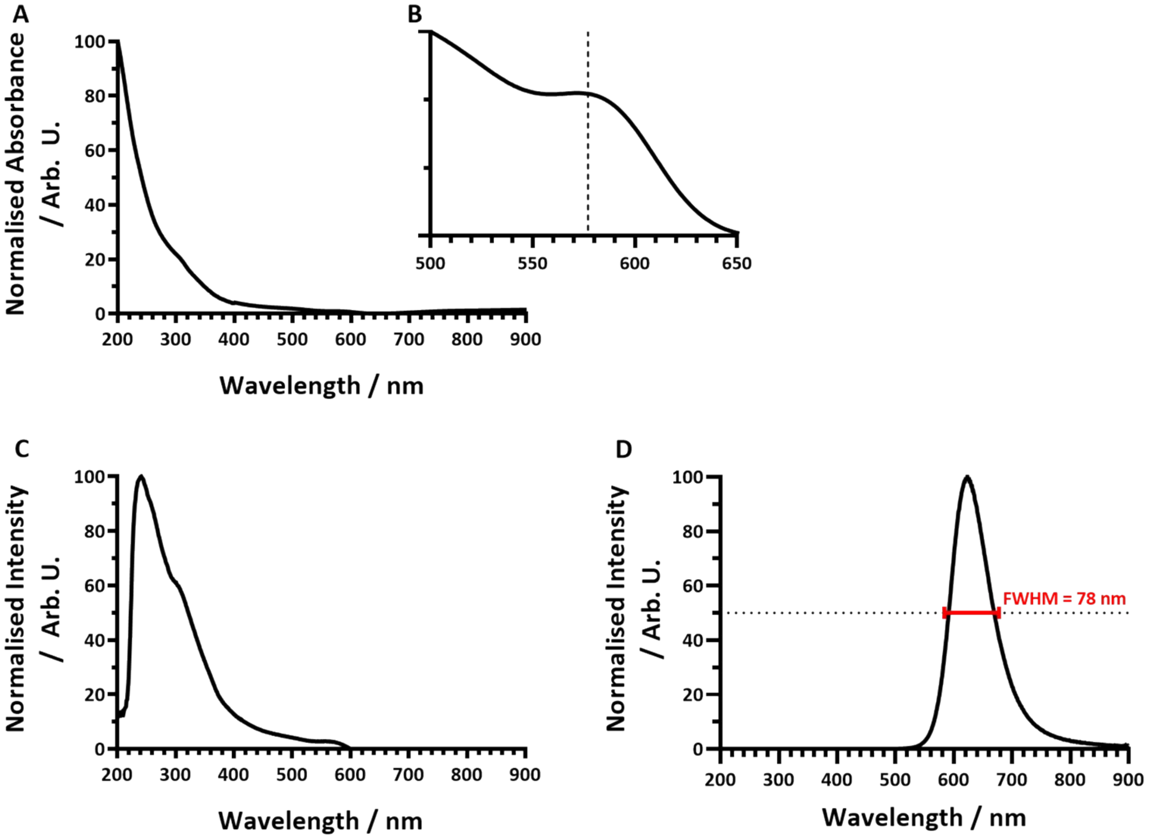

Figure S18 – Optical properties of QD-Pen-PEG. A) Absorbance. B) Expanded absorbance around 1st excitonic peak. C) Excitation spectrum, λ_{em} = 610 nm. D) Emission spectrum with FWHM indicated. λ_{ex} = 405 nm. Measurements performed in water at 25 °C.

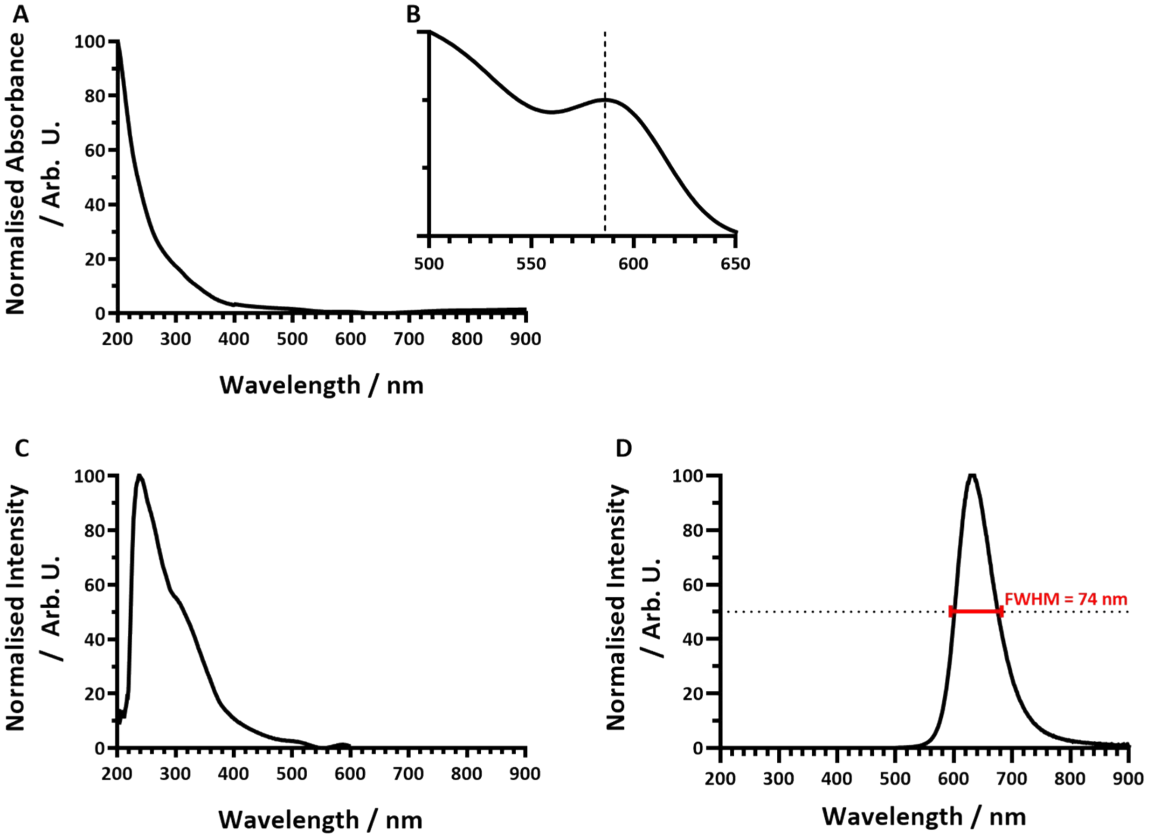

Figure S19 – Optical properties of QD-LA-PEG. A) Absorbance. B) Expanded absorbance around 1st excitonic peak. C) Excitation spectrum, λ_{em} = 610 nm. D) Emission spectrum with FWHM indicated. λ_{ex} = 405 nm. Measurements performed in water at 25 °C.

Table S4: Components of exponential tail fits of fluorescent lifetime decay.[a]

	τ_1		τ_2		τ_3		τ_4		χ^2
	**Value**	**%**	**Value**	**%**	**Value**	**%**	**Value**	**%**	
**QD-Pen**	4.5 ± 0.3	8.7	19.3 ± 0.8	39.9	60.3 ± 2.3	41.0	240.9 ± 13.0	10.5	1.1297
**QD-Pen-PEG**	5.9 ± 0.4	8.2	24.4 ± 1.1	40.2	68.1 ± 2.7	42.0	250.9 ± 13.7	9.6	1.1042
**QD-LA**	9.1 ± 0.7	6.3	36.2 ± 2.1	41.5	86.8 ± 5.2	13.7	273.8 ± 24.2	8.5	1.0935
**QD-LA-PEG**	9.6 ± 0.4	9.3	36.6 ± 1.2	45.5	90.6 ± 3.0	39.2	318.0 ± 17.3	6.0	1.1651

[a]measured in water at 25 °C. λ_{ex} = 405 nm, λ_{em}, = 610 nm.

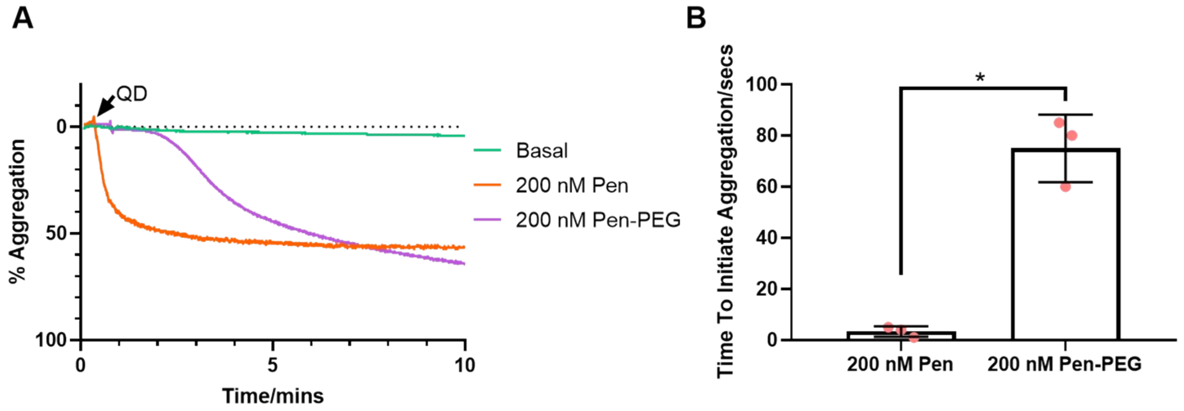

Figure S20 - PEGylation of QD reduces time to initiation of aggregation of QD-mediated platelet aggregation. Washed platelets at $2.5x10^8$ platelets/mL were incubated with either QD-Pen or QD-Pen-PEG (n=3) at 200 nM in stirring conditions at 37 °C for 10 min whilst aggregation was monitored with representative traces shown in (A) and time to initiate aggregation shown in (B). Data is shown as mean ± SD. n=3. Statistical significance was calculated using a t-test. *p≤0.05.

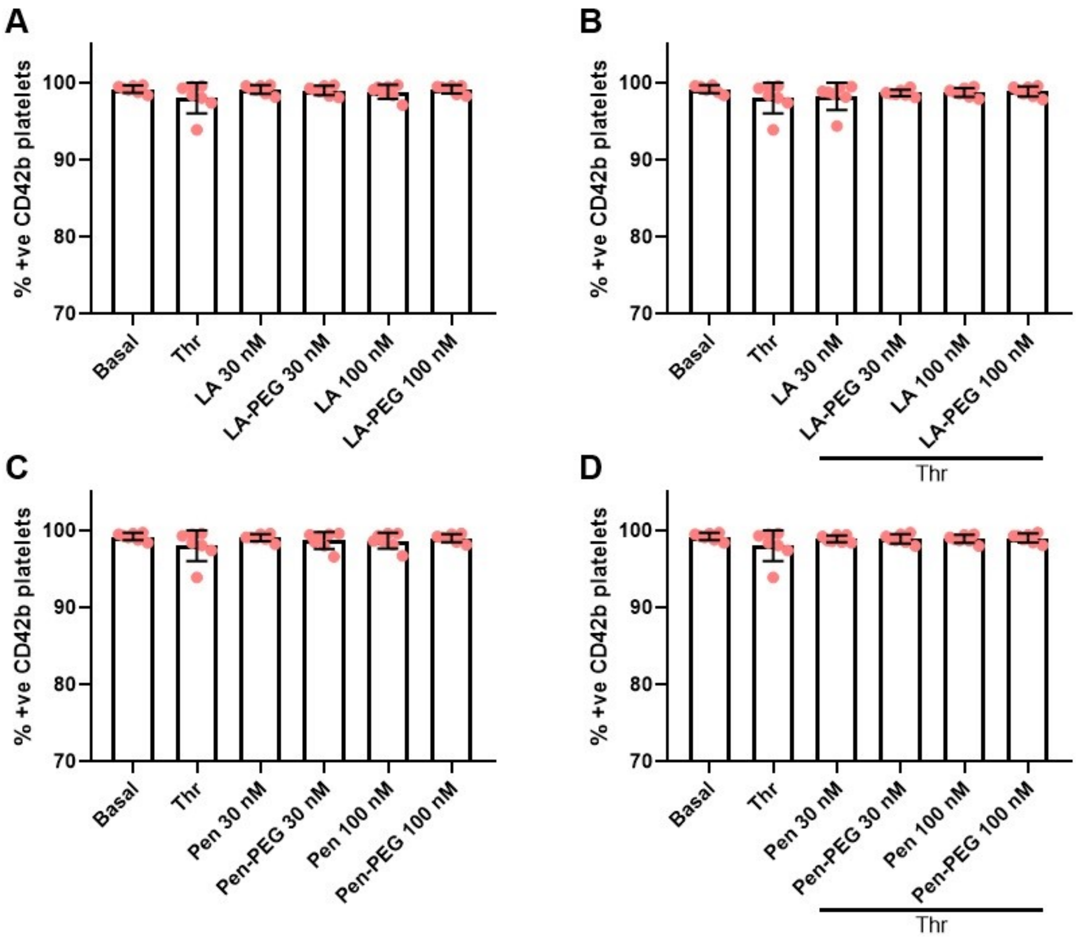

Figure S21 - CD42b Platelet Expression. Washed platelets at 1x10^7 platelets/mL were incubated with monoclonal antibodies against CD42b for 5 minutes. QD-LA, QD-LA-PEG, QD-Pen, or QD-Pen-PEG at either 30 nM or 100 nM for 20 min was then added to platelets, before the addition of either Tyrode's or 0.1U/ml thrombin before fixing with 0.45% PFA and being analysed by flow cytometry. Number of platelets positive for CD42b in (A) platelets incubated with QD-LA and QD-LA-PEG in basal conditions or (B) stimulated with thrombin, (C) platelets incubated with QD-PEN or QD-Pen-PEG in basal conditions or (D) stimulated with thrombin. Data is shown as mean ± SD. n=7. Statistical significance was calculated using a two-way ANOVA followed by a Sidak's multiple comparison post-hoc test. No statistical significance was found.

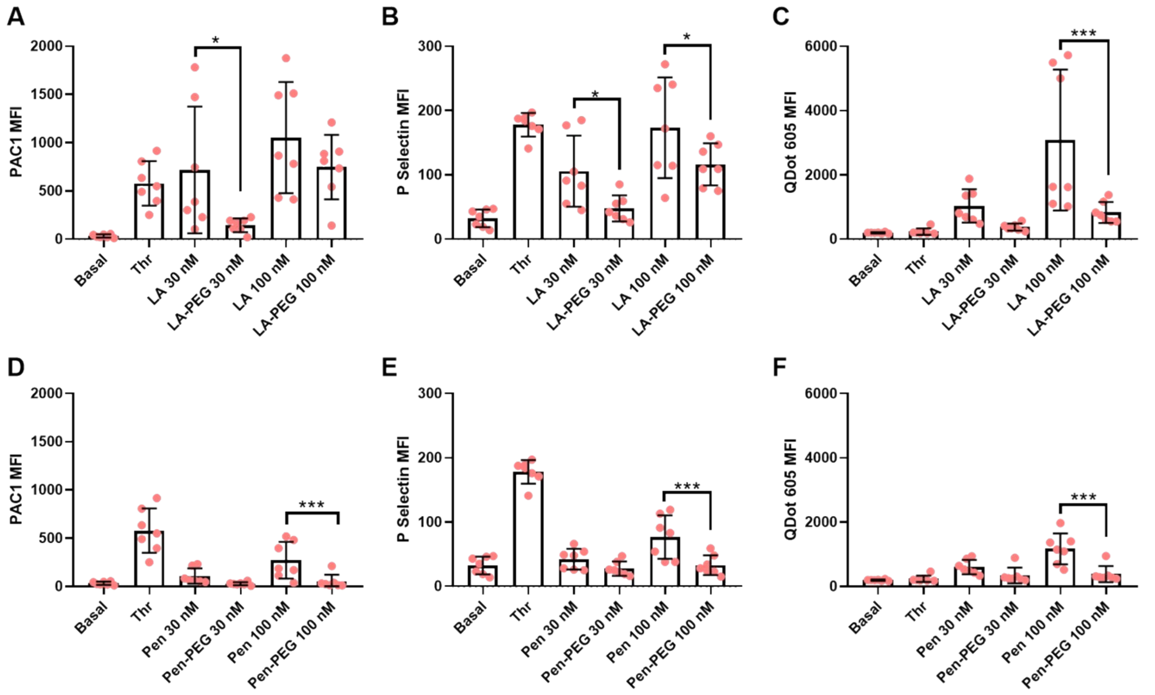

Figure S22 - Platelet QD-mediated activation is reduced by PEGylation of QDs. Washed platelets at $1x10^7$ platelets/mL were incubated with monoclonal antibodies against P-selectin, CD42b, and CD41/CD61 for 5 minutes before the addition of either QD-LA, QD-LA-PEG, QD-Pen, or QD-Pen-PEG at either 30 nM or 100 nM for 20 min before fixing with 0.45% PFA and being analysed by flow cytometry. MFIs for (A), (D) PAC1, and (B) (E) P-selectin) and (C) (F) QDs are shown. Data is shown as mean ± SD. n=7. Statistical significance was calculated using a two-way ANOVA followed by a Sidak's multiple comparison post-hoc test. *p≤0.05, **p≤0.01, ***p≤0.001.

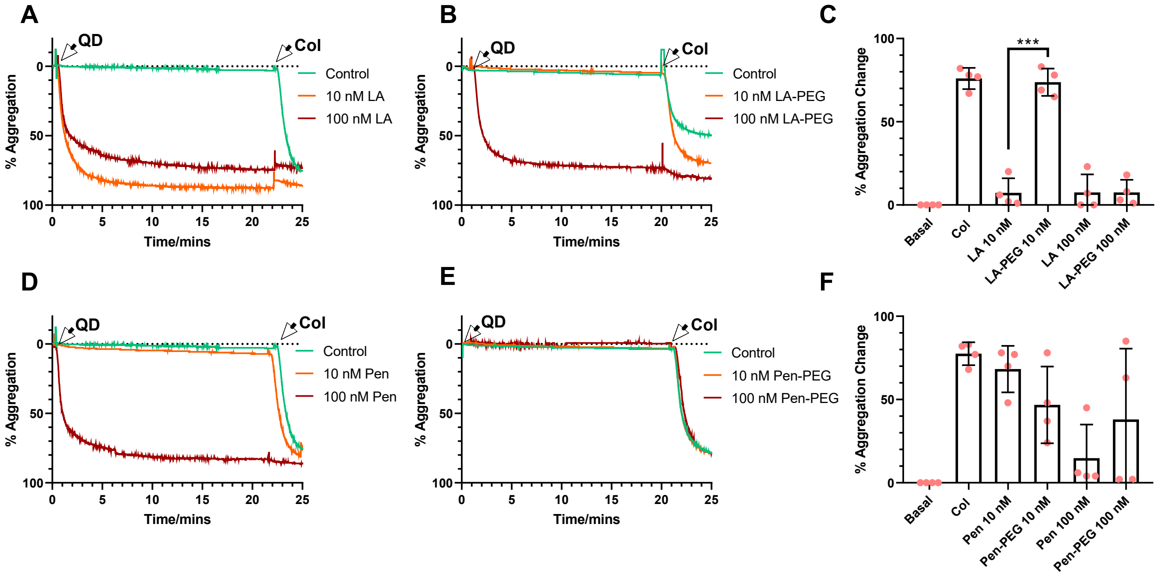

Figure S23 - PEGylation of QDs reserve platelets ability to aggregate to collagen. Washed platelets at 2.5x10^8 platelets/mL were incubated with either (A) QD-LA and (B) QD-LA-PEG, or (D) QD-Pen and (E) QD-Pen-PEG at either 10 nM or 100 nM in stirring conditions at 37 ^{o}C for 20 min before stimulation with 3 ug/mL collagen. Aggregation was monitored and representative traces shown. Final aggregation shown in (C) and (F). Data is shown as mean ± SD. n = 4. Statistical significance was calculated using a two-way ANOVA followed by a Sidak's multiple comparison post-hoc test. *p≤0.05, **p≤0.01, ***p≤0.001.

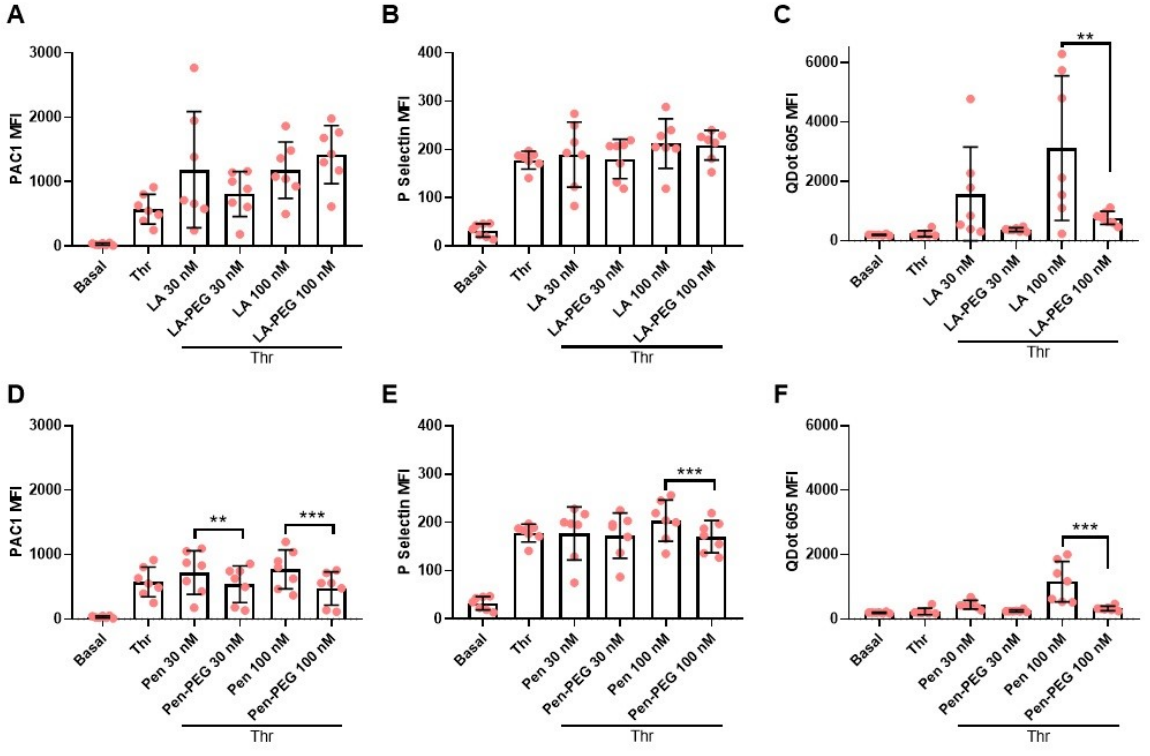

Figure S24 - Platelet activation markers to thrombin as still expressed in the presence of PEGylated QDs. Washed platelets at 1x10^7 platelets/mL were incubated with monoclonal antibodies against P-selectin, CD42b, and CD41/CD61 for 5 minutes before the addition of either QD-LA, QD-LA-PEG, QD-Pen, or QD-Pen-PEG at either 30 nM or 100 nM for 20 min. 0.1 U/mL thrombin was added to platelets for 10 minutes before fixing with 0.45% PFA and being analysed by flow cytometry. MFIs for (A), (D) PAC1, and (B) (E) P-selectin) and (C) (F) QDs are shown. Data is shown as mean ± SD. n=7. Statistical significance was calculated using a two-way ANOVA followed by a Sidak's multiple comparison post-hoc test. *p≤0.05, **p≤0.01, ***p≤0.001.

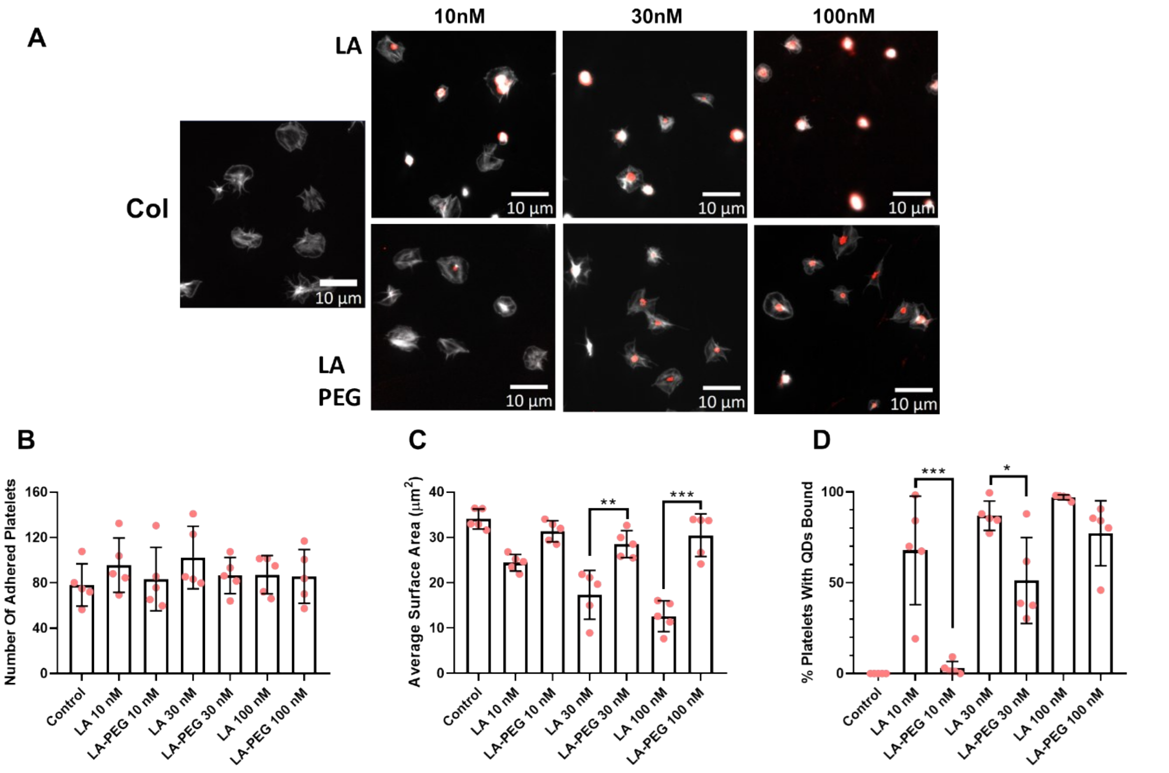

Figure S25 - Platelet spreading is maintained in the presence of PEGylated LA QDs. Washed platelets at 2x10^7 platelets/mL were spread on 100 µg/mL collagen (col) in the absence, or presence of either QD-LA or QD-LA-PEG at 10, 30 or 100 nM for 25 min before permeabilizing, fixing and staining with FITC-Phalloidin using an NA 1.4 oil x63 objective. Coverslips were mounted and visualized by fluorescence microscopy. (A) Representative images with scale bars showing 10 µm. Graphs show (B) number of adhered platelets, (C) average surface area of platelets (µm^2), and (D) percentage of platelets with QDs bound. Data is shown as mean ± SD. n=5. Statistical significance was calculated using a two-way ANOVA followed by a Sidak's multiple comparison post-hoc test. *p≤0.05, **p≤0.01, ***p≤0.001

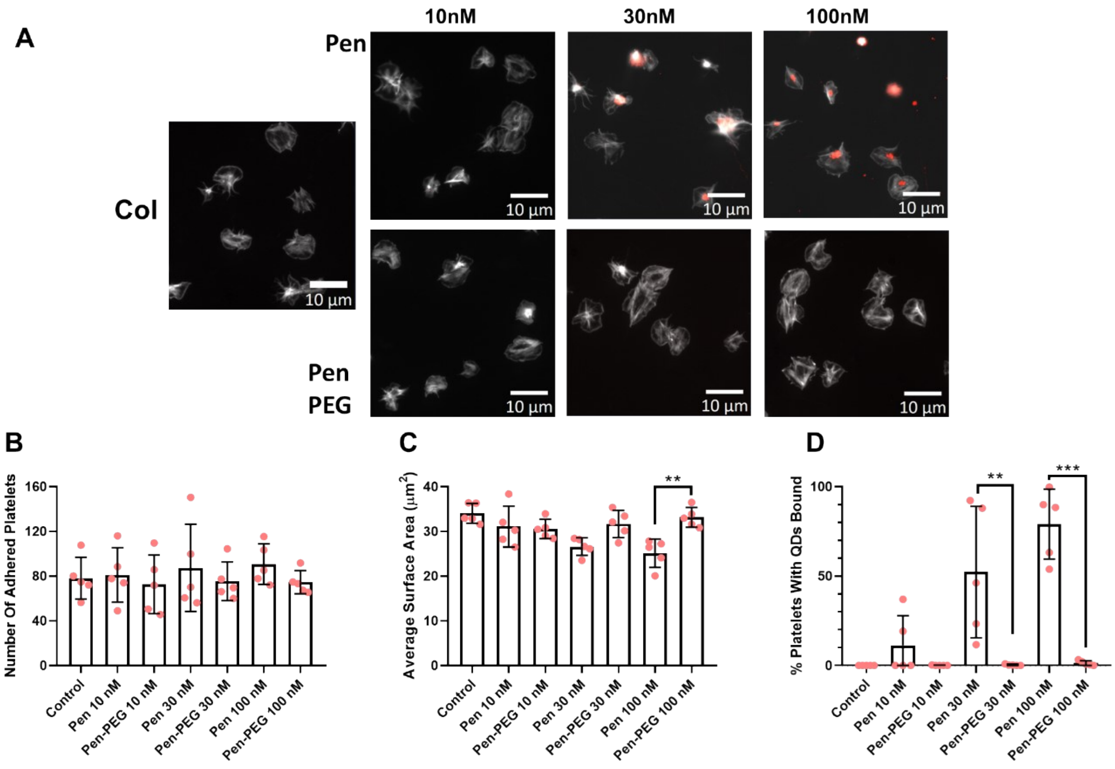

Figure S26 - Platelet spreading is maintained in the presence of PEGylated Pen QDs. Washed platelets at 2x10^7 platelets/mL were spread on 100 µg/mL collagen (col) in the absence, or presence of either QD-Pen or QD-Pen-PEG at 10, 30 or 100 nM for 25 min before permeabilizing, fixing and staining with FITC-Phalloidin. Coverslips were mounted and visualized by fluorescence microscopy using an NA 1.4 oil x63 objective. (A) Representative images with scale bars showing 10 µm. Graphs show (B) number of adhered platelets, (C) average surface area of platelets (µm^2), and (D) percentage of platelets with QDs bound. Data is shown as mean ± SD. n=5. Statistical significance was calculated using a one-way ANOVA followed by a Tukey's multiple comparison post-hoc test. *p≤0.05, **p≤0.01, ***p≤0.001

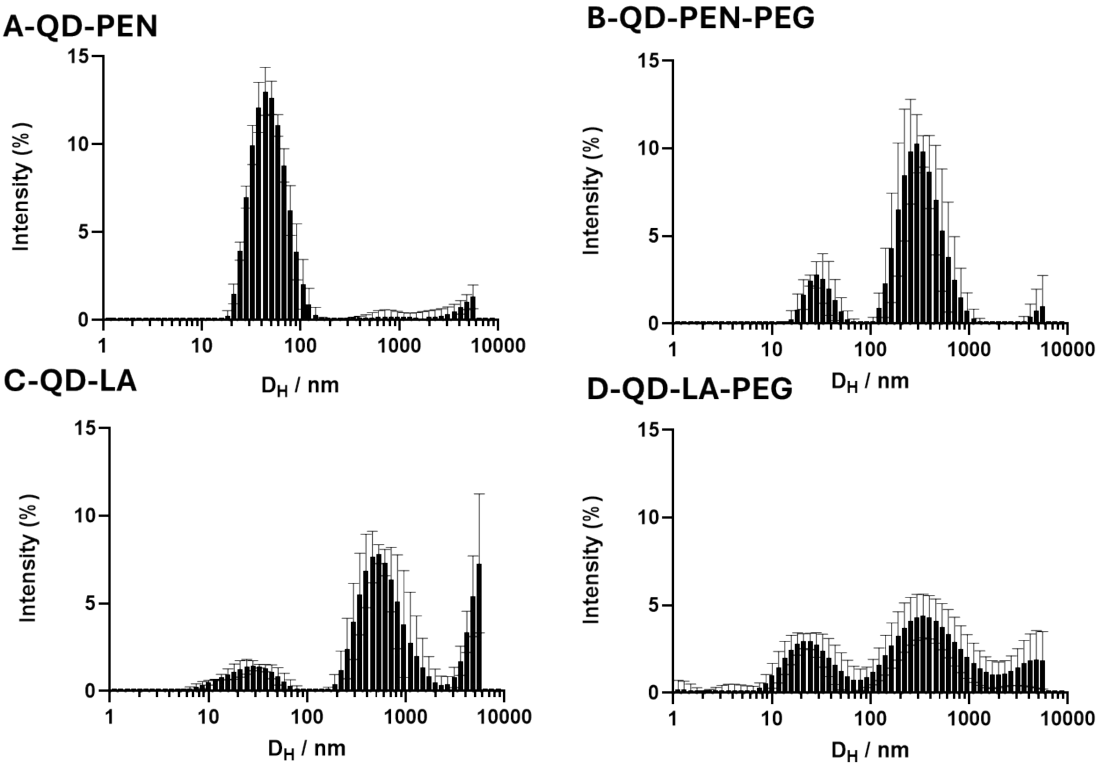

Figure S27: Histograms of DLS represented as intensity. A) QD-Pen, B) QD-Pen-PEG, C) QD-LA, D) QD-LA-PEG. Error bars indicate standard deviation. Measured at 25 °C in water. Peaks at higher diameters are due to the emissive nature of the QDs in the range corresponding to the instrument laser (~633 nm)[1] causing erroneous signals.[2] This is overemphasised in intensity representations of the data in comparison to number representations (Figure S10) due to the relationship between intensity and diameter (intensity is proportional to d^6, whereas number is proportional to d)[3] as described by the Rayleigh approximations.

References

[1] Technical Note: Measuring Quantum Dots Using the Zetasizer Nano, Malvern Panalytical, https://www.malvernpanalytical.com/en/learn/knowledge-center/application-notes/an101104measuringquantumdotszetasizernano, (Accessed November 2024)

[2] Technical Note: Measuring the size of fluorescent Quantum Dots using Zetasizer Advance, MAlvern PAnalytical, https://www.malvernpanalytical.com/en/learn/knowledge-center/application-notes/an220818-quantum-dots-zetasizer(Accessed November 2024)

[3] Technical Note:; Number and Volume Size Distributions, Malvern Panalytical, https://www.malvernpanalytical.com/en/learn/knowledge-center/application-notes/an140403numbervolumesizedistributions, (Accessed November 2024)
